# GapR binds DNA through dynamic opening of its tetrameric interface

**DOI:** 10.1093/nar/gkaa644

**Published:** 2020-08-05

**Authors:** Qian Huang, Bo Duan, Xianzhi Dong, Shilong Fan, Bin Xia

**Affiliations:** Beijing Nuclear Magnetic Resonance Center, School of Life Sciences, College of Chemistry and Molecular Engineering, Peking University, Beijing, 100871, China; Beijing Nuclear Magnetic Resonance Center, School of Life Sciences, College of Chemistry and Molecular Engineering, Peking University, Beijing, 100871, China; Institute of Biophysics, Chinese Academy of Science, Beijing 100101, China; The Technology Center for Protein Sciences, Tsinghua University, Beijing 100084, China; Beijing Nuclear Magnetic Resonance Center, School of Life Sciences, College of Chemistry and Molecular Engineering, Peking University, Beijing, 100871, China

## Abstract

GapR is a nucleoid-associated protein that is an essential regulator of chromosome replication in the cell cycle model *Caulobacter crescentus*. Here, we demonstrate that free GapR is a homotetramer, but not a dimer as previously reported (Guo *et al.*, Cell 175: 583–597, 2018). We have determined the crystal structure of GapR in complex with a 10-bp A-tract DNA, which has an open tetrameric conformation, different from the closed clamp conformation in the previously reported crystal structure of GapR/DNA complex. The free GapR adopts multiple conformations in dynamic exchange equilibrium, with the major conformation resembling the closed tetrameric conformation, while the open tetrameric conformation is a representative of minor conformers. As it is impossible for the circular genomic DNA to get into the central DNA binding tunnel of the major conformation, we propose that GapR initially binds DNA through the open conformation, and then undergoes structural rearrangement to form the closed conformation which fully encircles the DNA. GapR prefers to bind DNA with 10-bp consecutive A/T base pairs nonselectively (*K*_d_ ∼12 nM), while it can also bind GC-rich DNA sequence with a reasonable affinity of about 120 nM. Besides, our results suggest that GapR binding results in widening the minor groove of AT-rich DNA, instead of overtwisting DNA.

## INTRODUCTION

GapR (growth-associated A/T-binding protein involved in regulation) is a chromosome structure protein conserved in α-proteobacteria, and it is discovered initially as an essential nucleoid-associated protein for normal growth and cell division in *Caulobacter crescentus* (1–3). Depletion of GapR gene in *Caulobacter* cells results in severe defects in cell growth, cell division, cell size, DNA replication, chromosome segregation/condensation and gene expression (2,4,5).

GapR is found to accumulate at the origin of replication (*Cori*) in chromosomal DNA (4), while it displays asymmetric dynamic distribution (from *ori* to *ter*) in DNA replication, and has a gradual condensation ahead of replication forks (replication-eviction model) (5,6). GapR-depleted cells show significant defects for proper DNA replication initiation and elongation (2). It was reported that GapR can stimulate topoisomerases to relax positive supercoil of DNA during replication (7). GapR is found to affect global gene expression, while its effect is higher for genes located closer to the replication origin than those closer to the terminus region (5), correlating to the general bias in GapR enrichment along the chromosome (4). GapR is enriched in the promoter regions of cell cycle-regulated genes, but only displays a mild regulatory role on the transcription (2,4). Although GapR generally prefers to bind AT-rich DNA sequences (2,5), enrichment of GapR is also found at the 3′ end of highly expressed genes and operons, which have low AT contents (7).

**Figure 1.**
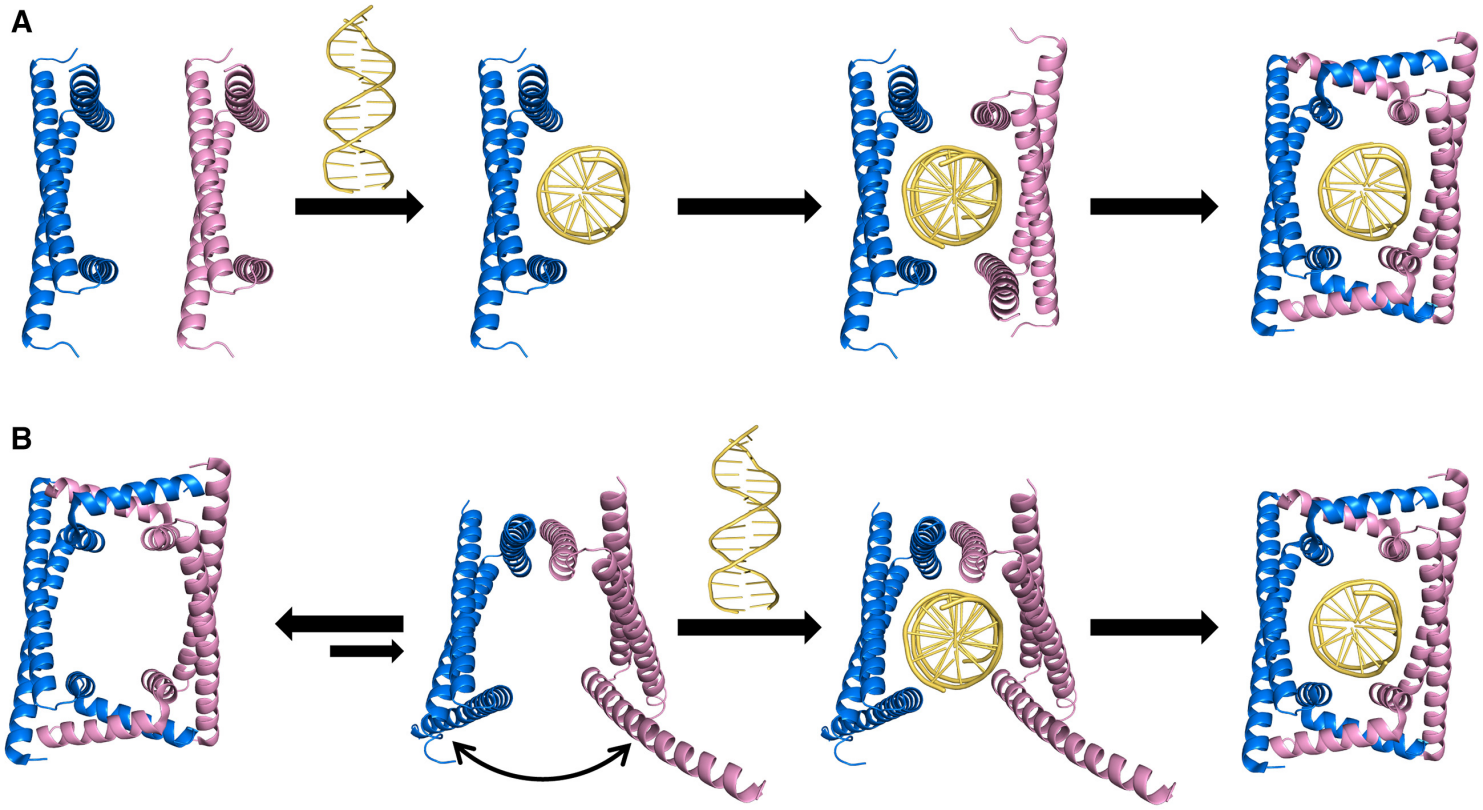
Comparison of the proposed models for GapR binding DNA. (**A**) The previously proposed model: The free GapR is dimeric. Two free dimeric GapR molecules bind the target sequence of DNA, and then form a closed tetramer to enclose the DNA inside its central tunnel (7). (**B**) The model proposed from this work: The free GapR is tetrameric. It is in equilibrium between the closed conformation and the open conformations. The open conformers can bind DNA, and then convert into the closed conformation stabilized by the bound DNA.

The crystal structures of the free GapR from *Bosea* sp. *Root381* and *C. crescentus* GapR (residue 11–89) in complex with an 11-bp AT-rich DNA have been reported (7). For the free GapR, the structure reveals a dimer with helices α1 of the two protomers form an antiparallel coiled-coil dimer interface, while the other helices α2 (residues 45–76) are protruding at opposite ends of the antiparallel coiled-coil domain. However, in the complex structure (PDB ID: 6CG8), GapR adopts a closed tetrameric conformation to encircle the DNA in a snug clamp conformation. While the coiled-coil dimer interface still retains in the tetramer, each helix α2 of the dimer is kinked and split into two α-helices, and interactions between α2 and α3 pairs result in the formation of the tetramer. It was suggested that GapR prefers to bind overtwisting AT-rich DNA, but not GC-rich DNA, due to the size limitation of its central tunnel positioning DNA. Based on these results, it was proposed that the GapR dimer tracks along the DNA as a caliper to search for sites with narrow minor and expanded major grooves, and then forms the closed tetrameric conformation with another GapR dimer to completely enclose the DNA (Figure 1A) (7). However, a recent study reported that GapR is a tetramer in solution, and the DNA binding does not alter its oligomeric state (8). It was suggested that GapR binds DNA non-specifically and should be able to accommodate B-DNA freely inside the central tunnel channel, which is wider in a new crystal structure of GapR (PDB ID: 6OZX) than that of the structure 6CG8.

In this study, we conclude that free GapR from *C*. *crescentus* is actually also a tetramer, but not a dimer as previously reported. We demonstrate that there exist minor conformers for free GapR in solution, which are in dynamic equilibrium with the major conformation resembling the previously reported closed tetrameric conformation. We report the crystal structure of a 10-bp A-tract DNA bound by GapR in an open tetrameric conformation, which is representative of the minor conformers of free GapR. We propose that GapR binds DNA through the minor open tetrameric conformation, which is then converted into the closed tetrameric conformation stabilized by the bound DNA (Figure 1B). Although GapR prefers to bind AT-rich DNA sequences nonselectively, it can also bind GC-rich DNA with high affinity.

## MATERIALS AND METHODS

### Protein expression and purification

The coding sequences of GapR and its truncation mutants were amplified from GapR gene (CCNA_03428) and inserted into the NdeI and XhoI sites of pET-21a(+) expression vector (Novagen), containing a C-terminal His_6_-tag coding sequence. For GapR^ΔN10^, pET-28a(+) expression vector was used, with a thrombin protease cleavable N-terminal His_6_-tag. The mutations of GapR were generated using the site-directed mutagenesis kit (SBS Genetech).

All the proteins were expressed in *Escherichia coli* Rosetta (DE3) cells. Bacteria were cultured in Luria-Bertani (LB) medium and expressed for 6 h at 35°C after induction with 0.1 mM IPTG (isopropylthio-β-d-galactoside). For the preparation of NMR samples, ^15^N- or ^13^C-labeled M9 minimal media were used instead of LB medium. Cells were lysed by brief sonication in 50 mM sodium phosphate buffer (pH 8.0) with 1 M NaCl, 20 mM imidazole and 1 mM PMSF. The cell lysate was centrifugated and the supernatant was applied onto a Ni-NTA column (Qiagen), and eluted with elution buffer (50 mM sodium phosphate, 1 M NaCl, 250 mM imidazole, pH 8.0). The protein was further purified with size-exclusion chromatography using a Superdex 75 column (GE Healthcare) equilibrated in 50 mM sodium phosphate buffer (pH 7.0) with 150 mM NaCl, while GapR protein samples used for crystallization were purified with 25 mM Tris buffer (pH 8.0) containing 300 mM NaCl. N-terminal His-tag of the GapR^ΔN10^ was removed by thrombin proteolysis after purification. The cleaved tag was removed with size-exclusion chromatography. The purity of GapR protein was estimated to be >95% based on SDS/PAGE.

To prepare double-strand DNA samples, hybridization of complementary DNA oligonucleotides was carried out by first heating to 94°C for 5 min, and then slowly cooling down to room temperature. For the preparation of GapR/DNA complex samples, GapR protein and double-strand DNA were first mixed together at ∼ 1:1 molar ratio, and the complex was purified with size-exclusion chromatography.

### Chemical cross-linking

Protein (0.1 mM) was cross-linked with EGS (ethylene glycol bis-(succinimidyl succinate)) (Pierce) in the reaction buffer (50 mM sodium phosphate, 150 mM NaCl, pH 7.2). The reaction mixtures were incubated at room temperature for 10 min, with two EGS concentrations (0.5 mM and 1 mM), and then the reaction was quenched by adding 1M Tris–HCl (pH 7.5) buffer to a final concentration of 100 mM.

### Analytical ultracentrifugation (AUC)

Sedimentation velocity experiments were performed with a Proteomelab XL-I analytical ultracentrifuge (Beckman Coulter, Brea, CA) equipped with AN-60Ti rotor (4-holes) and absorbance optics, at a speed of 52 000 rpm at 20°C. GapR^ΔC17^ (concentration 4.0 mg/ml) and GapR (concentrations 1.0, 2.1 and 3.3 mg/ml) protein samples were in 50 mM sodium phosphate buffer (pH 7.0) with 150 mM NaCl. Radial absorbance scans were collected at 280 nm at a spacing of 3 mm with three average in a continuous scan mode. The differential sedimentation coefficients, *c*(*s*) and molecular weight were calculated using SEDFIT software (9).

### Size exclusion chromatography and multi-angle light scattering (SEC-MALS)

SEC-MALS was performed by first passing the protein sample through a Superdex-75 HR 10/30 size exclusion chromatography column (GE Healthcare) at a flow rate of 0.5 ml/min, using AKTA Pure Liquid Chromatography System (GE Healthcare), and the eluted protein fraction was monitored with a DAWN^®^ HELEOS™ light scattering detector (Wyatt). The SEC-MALS system was equilibrated with 50 mM sodium phosphate buffer (pH 7.0) containing 150 mM NaCl. GapR (concentrations 0.5, 1.0 and 2.0 mg/mL), GapR^ΔC17^ (concentration 2.0 mg/ml) and GapR/DNA samples (concentration 2.0 mg/ml) were injected into the system at 25°C. Data acquisition and analysis were carried out using the ASTRA software (Wyatt).

### Isothermal titration calorimetry (ITC)

ITC experiments were performed on a MicroCal PEAK system (Malvern) at 4°C. Protein and DNA samples were prepared in 50 mM sodium phosphate buffer (pH 7.0) with 200 mM NaCl. 25 μM tetrameric GapR protein was placed in the cell, and DNA (0.25 mM) was injected in 2.0-μl aliquots. Corresponding DNA to buffer titration experiments were performed as controls. ITC titration data were analyzed using the MicroCal PEAQ-ITC software. Each measurement is repeated twice, with one of the curves shown in the figures.

### NMR data collection

All NMR samples of GapR were in 50 mM sodium phosphate buffer (pH 7.0) containing 150 mM NaCl, prepared in 90% H_2_O/10% D_2_O with 0.01% DSS. Except for the temperature-dependent experiment, all the other NMR experiments were performed at 298 K.

For DNA titration experiments, a series of 2D ^1^H–^15^N HSQC or 2D ^1^H–^13^C HSQC spectra for tetrameric GapR (0.1 mM) were collected with the addition of DNA at final concentrations of 0.02, 0.04, 0.06 and 0.1 mM, respectively. All of the experimental data were collected on Bruker AVANCE 800 or 950 MHz spectrometers with a triple-resonance TCI cryoprobe. Proton chemical shifts were referenced directly to internal DSS. ^15^N and ^13^C chemical shifts were referenced indirectly to DSS.

For 1D ^1^H NMR experiments to monitor the imino protons of DNA, water suppression for sample in 90% H_2_O/10% D_2_O was achieved using combined excitation sculpting with gradients and additional flip-back pulse (10).

### Protein crystallization

GapR/10A complex sample was obtained by co-purification of GapR protein with DNA duplex (5′-CCGAAAAAAAAAACGC-3′) using SEC and concentrated to 8.0 mg/ml in 25 mM Tris buffer (pH 8.0) containing 150 mM NaCl. Crystals of WT GapR and its selenomethionyl derivative in complex with DNA were obtained at 298 K by sitting drop vapor diffusion using an equal volume of protein/DNA complex sample and crystallization solution consisting of 0.01 M manganese(II) chloride tetrahydrate, 0.1 M sodium citrate (pH 5.6) and 2.5 M 1,6-hexanediol. Crystals of free GapR were obtained by mixing an equal volume of complex sample and crystallization solution consisting of 0.01 M manganese (II) chloride tetrahydrate, 0.1 M sodium citrate (pH 5.6), 2.5 M 1,6-hexanediol and 0.3% (v/v) dimethyl sulfoxide. Crystals were frozen in liquid nitrogen after a quick soak in a cryo-protectant comprising crystallization solution with 25% glycerol for data collection.

### Crystallographic structure determination

All X-ray diffraction data were collected at beamline BL17U at Shanghai Synchrotron Radiation Facility (Shanghai, China). Both the native and MAD datasets were integrated and scaled using HKL2000 software (HKL Research, Inc.). The space group identified for free GapR was *P*3_1_21, while the GapR/10A complex was P65. The complex structure was determined by the multiple wavelength anomalous dispersion phasing method (MAD) with a selenomethionyl crystal. The program PHENIX Autosol was used to locate the heavy atoms and calculate the initial phases, leading to an interpretable electron density map. The initial models were built automatically with the program Autobuild in PHENIX. The manual model building was carried out by Coot (11). The model was then refined with the PHENIX program (12). Finally, a model of GapR/10A complex refined to *R*_work_/*R*_free_ values of 22.1/26.8% was obtained, at 2.4 Å resolution. Data collection and the final model statistics are summarized in Table 1. Using the complex structure as the search model, the GapR dimer structure was determined with molecular replacement and refined with the same procedure. The final model converged to *R*_work_/*R*_free_ values of 20.3/21% to 2.0 Å resolution. Data collection and structure refinement statistics were also listed in Table 1. All structure figures were generated using PyMOL or MOLMOL.

**Table 1.** Data collection and refinement statistics for the open GapR/DNA complex structure and the dimeric GapR structure.

	Open complex	Dimeric GapR
**Data collection**		
PDB code	6K2J	6JYK
Space Group	*P*65	*P*3_1_21
Cell dimensions		
*a*, *b*, *c* (Å)	65.3, 65.3, 274.2	55.0, 55.0, 111.0
α, β, γ (°)	90, 90, 120	90, 90, 120
Resolution (Å)	48.1–2.40	43.8–2.00
*R*_merge_ (%)	6.8 (94.4)	7.3 (54.0)
*I*/σ*I*	51.2 (2.2)	37.7 (3.5)
Completeness (%)	99.9 (99.8)	98.1 (100)
Redundancy	20.3 (18.6)	5.6 (6.6)
**Refinement**		
Resolution (Å)	48.1–2.40	43.8–2.00
No. reflections	25730	13454
*R*_work_/*R*_free_ (%)	22.1/26.8	20.3/21.0
R.m.s. deviations		
Bond lengths (Å)	0.004	0.009
Bond angles (°)	0.677	0.891
Ramachandran plot statistics (%)		
Most favoured	98.4	100
Additional allowed	1.6	0.0
Generously allowed	0.0	0.0
Disallowed	0.0	0.0

Values in parentheses are for the highest resolution shell. *R*_merge_= Σ_h_Σ_i_|*I_h,i_*-*I_h_*|/Σ_h_Σ_i_*I*_*h,i*_, where *I*_*h*_ is the mean intensity of the *i* observations of symmetry related reflections of *h*. *R* = Σ|*F*_obs –_ *F*_calc_|/Σ*F*_obs_, where *F*_calc_ is the calculated protein structure factor from the atomic model. *R*_free_ was calculated with 5% of the reflections selected randomly.

### Structure and interface analysis

Electrostatic potential surface was computed using APBS (13). Protein-protein or protein–DNA interfaces were analyzed using PISA at the European Bioinformatics Institute (14). DNA geometry parameters were analyzed using the Curves+ program (15).

### Small-angle X-ray scattering (SAXS)

Solution SAXS experiments were performed at the National Center for Protein Science Shanghai using the BL19U2 beamline at 298 K. A Pilatus 1M detector (DECTRIS Ltd) was used to record the scattered X-rays at a wavelength of 1.033 Å. Twenty consecutive frames of 1-second exposure time were recorded and averaged and have no difference between the frames. Scattering data of GapR (concentrations 1.0 and 2.0 mg/mL), GapR^ΔC17^ (concentration 1.0 mg/ml) and GapR/DNA samples (concentration 1.0 mg/ml) were collected in 50 mM sodium phosphate buffer (pH 7.0) containing 300 mM NaCl. SAXS profile for the buffer was recorded for background subtraction. The analysis of SAXS data was performed using ATSAS software package (16). Experimental *R*_g_ values were calculated from Guinier plot of the SAXS curves using the program PRIMUS in the ATSAS software package.

Any missing residues of GapR protein in the protein/DNA complex structures were patched using PyMOL, and the energy minimization was conducted to optimize the structures using AMBER 12 (17). The free tetrameric GapR protein structures were generated by removing the coordinates of DNA from the protein/DNA complex structures, followed by energy minimization using AMBER 12. The calculation of the theoretical scattering profiles and the fit of crystal structures to the experimental scattering profiles were performed using CRYSOL in the ATSAS software package. GAJOE program was used to select an ensemble of conformers at different percentages from a conformer pool, of which the ensemble-averaged scattering profile fits best to the experimental SAXS profile.

## RESULTS

### Free GapR is tetrameric in solution

**Figure 2.**
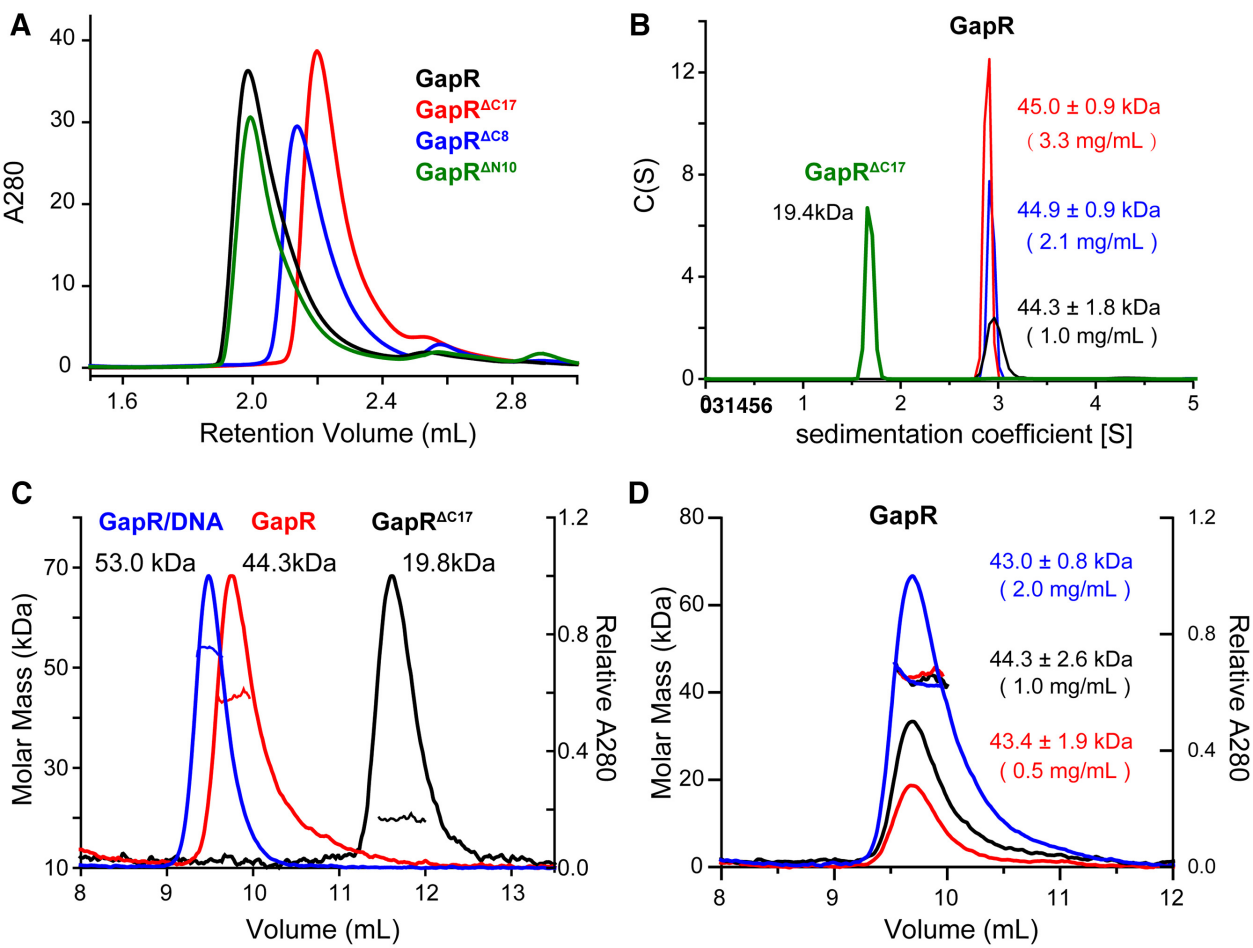
Free GapR is a tetramer. (**A**) Elution profiles from size-exclusion chromatography (SEC) for full-length GapR and its truncation mutants GapR^ΔC17^, GapR^ΔC8^, GapR ^ΔN10^. (**B**) AUC sedimentation analysis of GapR^ΔC17^ (concentration 4.0 mg/ml) and GapR (concentrations 1.0, 2.1 and 3.3 mg/ml). The calculated molecular weight values are indicated. (**C**) SEC-MALS analysis of GapR (concentration 1.0 mg/ml), GapR^ΔC17^ (concentration 2.0 mg/ml) and GapR/10A complex (concentration 2.0 mg/ml). Curves for molar mass (g/mol) vs. elution time (min) are shown, with calculated molecular weight values indicated. (**D**) SEC-MALS analysis of GapR at different concentrations (concentrations 0.5, 1.0 and 2.0 mg/ml).

When we started the biochemical characterization of GapR, no structural information was available. We first overexpressed GapR (residues 1–89) from *C. crescentus* in *E. coil*, and the recombinant protein was purified to homogeneity. On the size exclusion chromatography (SEC) column, GapR was eluted as a single peak, with an estimated molecular weight of 56.6 kDa (Figure 2A; Supplementary Table S1). This corresponds to approximately five times of the theoretical molecular mass (11.2 kDa) of GapR monomer, which is different from the previous report that free GapR is a dimer (7). Chemical cross-linking analysis with EGS reagent showed that the protein bands mostly migrate at positions corresponding to tetrameric and dimeric GapR (Supplementary Figure S1). To determine the molecular weight of free GapR more accurately, we performed analytical ultracentrifugation (AUC) and size-exclusion chromatography coupled to multi-angle light scattering (SEC-MALS) analysis. The measured molecular weights (∼44 kDa) of free GapR in solution are consistent with the theoretical molecular weight for a tetramer (44.8 kDa) (Figure 2B and C). Besides, SEC-MALS profile of GapR in complex with a 16-bp A-tract DNA d(5′-CCGAAAAAAAAAACGC) (10A) revealed a single peak with a molecular weight of 53.0 kDa (Figure 2C), matching the theoretical molecular weight for one tetrameric GapR and one DNA molecules (54.6 kDa). Together, these results clearly demonstrate that free GapR is tetrameric in solution, and there is no indication at all for free GapR to be a dimer, which also agrees with that of the recent report by Tarry *et al.* (8).

**Figure 3.**
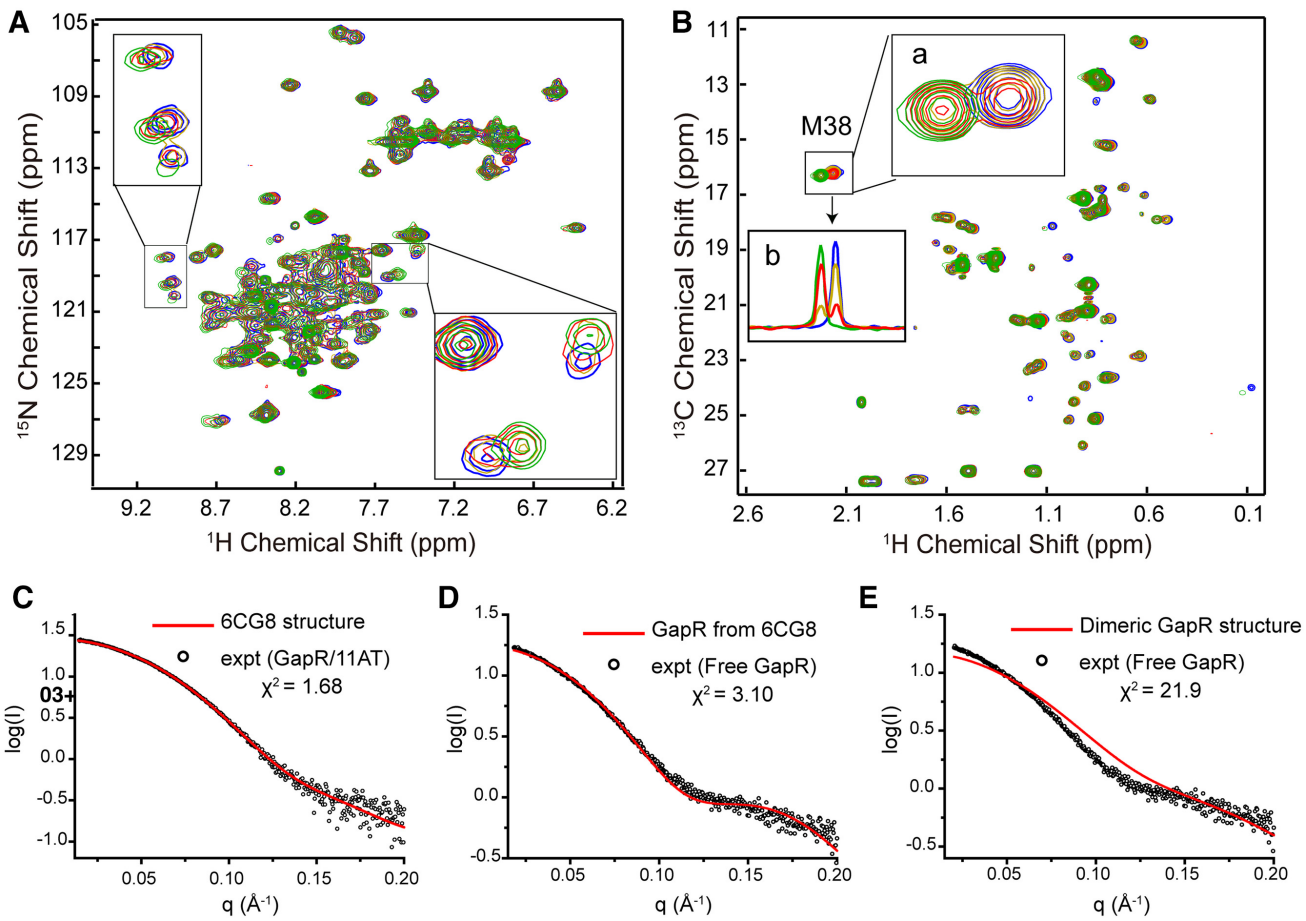
The conformation of free GapR tetramer resembles that in the GapR/DNA complex. (**A**) Overlay of 2D ^1^H–^15^N HSQC spectra of free GapR (blue) and with the addition of 10A DNA at DNA/protein ratios of 0.2 (gold), 0.6 (red) and 1.0 (green). (**B**) Overlay of 2D ^1^H–^13^C HSQC spectra of free (blue) GapR and with the addition of 10A DNA at DNA/protein ratios of 0.2 (gold), 0.6 (red) and 1.0 (green). Inserted boxes show an enlargement (a) or 1D ^1^H slices (b) of the signals from the methyl group of M38. (**C**) Comparison of experimental SAXS curve (black circle) of GapR/11AT complex and calculated scattering curve (red) based on the crystal structure 6CG8. (D, E) Comparison of experimental SAXS curve (black circle) of free GapR and the calculated scattering curve (red) based on the tetrameric GapR structure from 6CG8 (**D**) or the dimeric structure of free GapR (**E**).

We then used NMR to monitor the interaction between GapR and DNA. When 10A DNA was titrated into ^15^N labeled GapR sample, most NH signals in the 2D ^1^H-^15^N HSQC spectrum were not obviously perturbed, while about 10 of the NH signals of free protein became weaker, with new peaks showed up nearby, barely separated from the corresponding signals of the free protein (Figure 3A). This is the characteristic of slow exchange in the NMR time scale. In the previously reported crystal structure of GapR/DNA complex (PDB ID: 6CG8) (7), side-chains of residue M38 from the four protomers are all positioned inside the DNA binding core, with their methyl groups near the bound DNA. 2D ^1^H–^13^C HSQC NMR spectroscopy revealed that there is only one methionine methyl signal which also exhibits slow exchange characteristics during DNA titration, with the two peaks for free and DNA bound states very close to each other (Figure 3B). Some other methyl signals also showed similar patterns. What matters most is that the NMR signal differences between free and DNA bound states are very small in both the 2D ^1^H–^15^N HSQC and 2D ^1^H–^13^C HSQC spectra, suggesting that the DNA binding does not have a significant perturbation to the structure of GapR, and conformations of the free and DNA bound GapR should be mostly the same. It is worth noting that the NMR samples of free GapR tend to precipitate, and the precipitation is visible inside the NMR tube after a few hours at room temperature. However, no precipitation could be seen for over 24 h for the NMR samples of DNA bound GapR.

In the crystal structure of 6CG8, the N-terminal 10 residues were removed for the GapR protein, and the C-terminal tail and helix α3 (residues 69–89) make critical contribute for GapR to form the tetramer. We thus generated an N-terminal truncation mutant GapR^ΔN10^ (residues 11–89), and two C-terminal truncation mutants GapR^ΔC17^ (residues 1–72), GapR^ΔC8^ (residues 1–81). SEC analysis indicated that GapR^ΔN10^ has an apparent molecular weight of 50.0 kDa, also corresponding to approximately five times monomeric molecular mass, similar to that of full-length GapR. A comparison of 2D ^1^H–^15^N HSQC spectra reveals that most NH signals of GapR^ΔN10^ overlap well with those of full-length GapR (Supplementary Figure S2A). Thus, the N-terminal 10-residue truncation has little effect on the oligomerization of GapR, and GapR^ΔN10^ should have essentially the same structure as full-length GapR. However, the apparent molecular weights for GapR^ΔC17^ and GapR^ΔC8^ are 26.5 kDa and 32.9 kDa, respectively, much smaller than that of full-length GapR (Supplementary Table S1). NH signals in the 2D ^1^H–^15^N HSQC spectrum of ^ΔC17^ are distinct from those of full-length GapR (Supplementary Figure S2B), but can overlap well with those of GapR^ΔC8^ (Supplementary Figure S2C), suggesting that C-terminal deletions result in significant structural changes for GapR, while GapR^ΔC17^ and GapR^ΔC8^ should have essentially the same structure. Chemical cross-linking results showed that GapR^ΔC17^ protein band is at the position of a dimer (Supplementary Figure S1), with a theoretical molecular weight of 18.6 kDa. The molecular weight values for GapR^ΔC17^ determined using AUC and SEC-MALS are 19.4 and 19.8 kDa, respectively (Figure 2C and D). These results indicate that only the C-terminal deletions indeed have a dramatic impact on the oligomerization state of free GapR, consistent with the GapR structure in the crystal structure of 6CG8.

Small angle X-ray scattering (SAXS) was used to analyze the solution structures of free GapR and its complexes with 10A DNA, as well as DNA d(5’-TTAAAATTAAA) (11AT) which is the DNA duplex used in the crystal structure 6CG8. Guinier analysis of the SAXS data revealed that the radii of gyration (Rg) for GapR/10A and GapR/11AT are 26.8 and 26.7 Å, respectively, very close to the calculated *R*_g_ (26.3 Å) based on the crystal structure 6CG8. Fitting the calculated scattering curve of the GapR/11AT crystal structure to the experimental data yield a χ^2^ value of 1.68 (Figure 3C), indicating that the structure in solution is consistent with the crystal structure (18). On the other hand, the experimental *R*_g_ value for free GapR is 28.6 Å, a bit larger than those of GapR/DNA complexes. The fitting χ^2^ value is 3.10 between the experimental SAXS data of the free GapR and the calculated scattering curve based on the GapR protein structure from the crystal structure 6CG8 (Figure 3D), suggesting that the conformation of free GapR in solution should be similar to the tetrameric structure in GapR/11AT complex, in terms of size and shape.

Taken all together, we conclude that GapR is tetrameric in both free and DNA bound states, and the structure of tetrameric free GapR should be similar to that in the crystal structure of GapR/DNA complex. However, if free GapR also exists as a fully closed clamp conformation with a central tunnel to position the DNA, this raises the question that how the DNA could get into the central tunnel of GapR, as it is impossible for the circular *Caulobacter* genome DNA to thread inside the tunnel.

### Structure of GapR/10A complex reveals an open conformation

**Figure 4.**
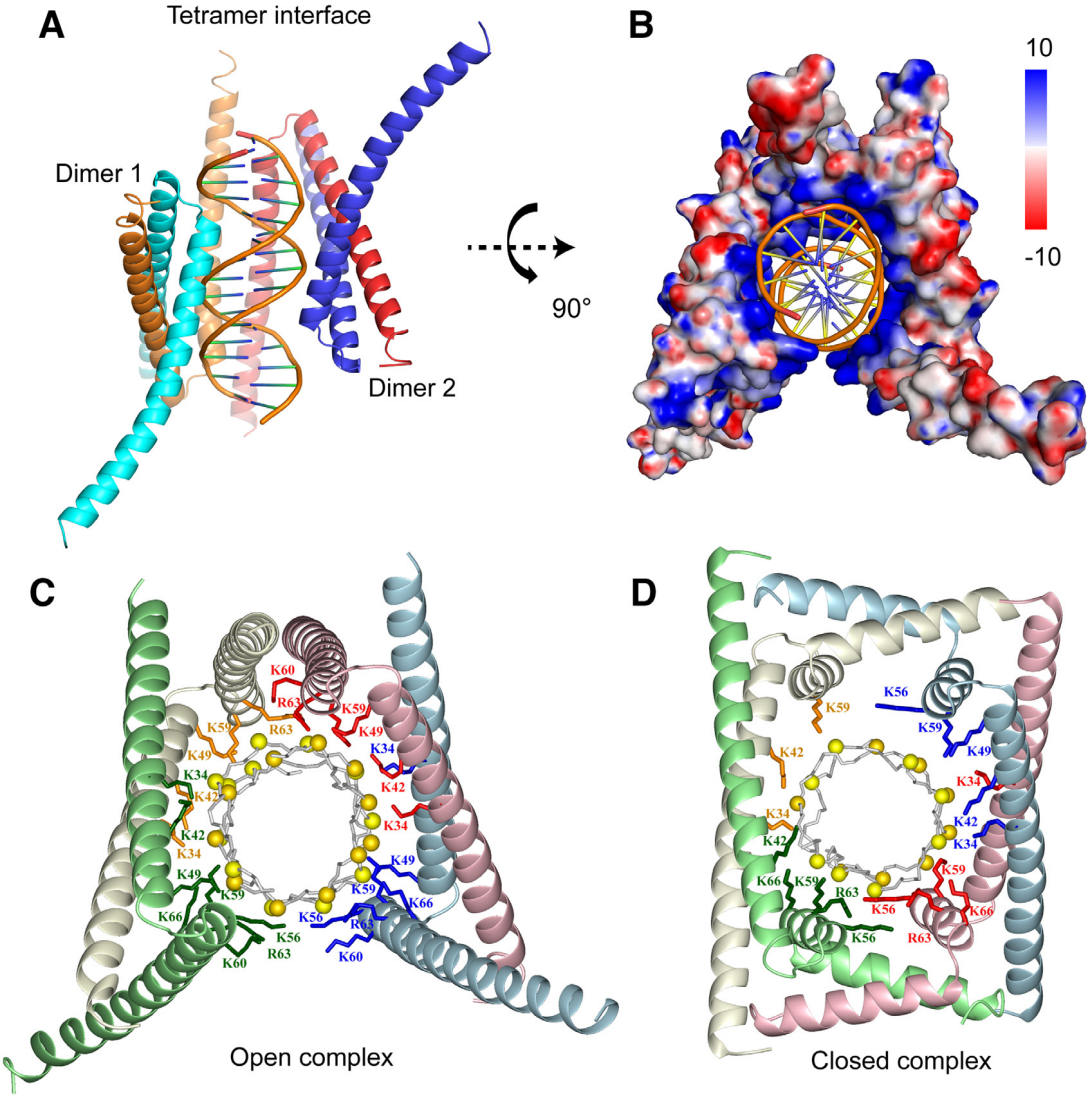
Crystal structure of GapR and 10A DNA complex reveals an open tetrameric conformation. (**A**) Ribbon diagram of GapR/10A complex structure (PDB ID: 6K2J). The four protomers are colored red, blue, orange, and cyan, respectively. DNA backbones are illustrated in gold. (**B**) Electrostatic potential surface of GapR protein in GapR/10A complex structure. (C, D) Ribbon diagrams of GapR and DNA complexes in open tetrameric conformation (**C**) or closed tetrameric conformation (PDB ID: 6CG8) (**D**) with DNA interacting lysine and arginine side-chains shown. The backbones of four protomers are colored in pink, light blue, pale green and ivory, respectively. The corresponding side-chains from each protomer are colored red, blue, green and orange, respectively. The phosphorus atoms of DNA are indicated in yellow.

Before the crystal structure 6CG8 was published, we have determined a crystal structure of GapR/10A complex, in which the tetrameric GapR adopts an open conformation, different from that in the crystal structure 6CG8 which is in a closed tetrameric conformation (Figure 4A and B). A summary of the crystallographic data statistics is shown in Table 1.

In this crystal structure, each protomer of GapR is consisted of two α-helices (α1: residues 13–51; α2: residues 55–89) that adopt a V-shape conformation. The open tetramer of GapR can be considered as a dimer of two antiparallel coiled-coil dimer-units formed mainly through α1 helices. The interface of each dimer-unit is identical to those in the structure of 6CG8, consisted of hydrophobic residues A16, L20, I23, I24, V27, L30, I37, I41, V44, A48, F53, L58, V61 and V62, with six intermolecular salt bridges formed between residues R26 and E47, E28 and R65, E31 and K66 (Supplementary Figure S3A). The two dimer-units form a tetramer through further dimerization with one α2 helix from each dimer-unit in an antiparallel coiled-coil conformation, while the other two α2 helices are free of contact. The tetrameric interface is formed by hydrophobic residues V57, V61, I64, I78, L81 and Y82, with 6 intermolecular salt bridges between residues K60 and E74, R63 and E74, D68 and K71 (Supplementary Figure S3B).

The 10A DNA is partially wrapped inside the open tetramer of GapR, which adopts a C-clamp like shape (Figure 4A–C). This is distinct from the crystal structure 6CG8, in which the 11AT DNA is fully enclosed inside the central tunnel of the closed tetramer (Figure 4D) (7). The two dimer-units in the open tetramer contact different portions of the DNA with virtually the same positively charged surfaces as those in the closed tetramer. The binding of 10A DNA is mainly through electrostatic interactions between the phosphate groups of a 15-bp DNA portion and side-chains of positively charged residues of GapR, consisting of K34, K49, K59 and R63 of all four protomers, K42 and K60 from three of the protomers, and K56 and K66 from two of the protomers (Figure 4C; Supplementary Figure S4A), according to interface analysis using PISA (14). There are no base-specific contacts between GapR and 10A DNA. For comparison, in GapR/11AT complex structure of the closed conformation, the DNA contacting residues are K59 of four protomers, K34, K42 and K56 from three protomers, R63 and K66 from three protomers, and K49 from only one protomer (Figure 4D; Supplementary Figure S4B).

While the two dimer-units of the tetrameric GapR in the open conformation adopt the same structure, the conformations of the two protomers in each dimer-unit are somewhat different mainly at residues 66–89 region of α2 helix, presumably due to that only one α2 helix from each dimer-unit participates in the tetrameric interface (Supplementary Figure S3C). The two protomers have a backbone heavy atom root-mean-square deviation (RMSD) of 0.29 Å for residues 16–65, with the helices for residues 66–89 region bent towards different directions. The conformation of the dimer-unit resembles that of the previously reported structure (PDB ID: 6CFY) of free GapR from *Bosea* sp. *Root381* (7), e.g. residues 55–89 form one long α-helix, while it split into two α-helices linked by a short loop of residues 67–68 to form the tetrameric interfaces in the closed tetrameric conformation. Therefore, the major difference between the two tetramer structures lies that one tetrameric interface of the closed conformation is fully dissociated in the open tetrameric conformation, while the other one is rearranged to form a new antiparallel coiled-coil interface (Figure 4C and D).

Interestingly, during our crystallization of GapR/10A sample, we also got a crystal which produced a dimeric structure of the *Caulobacter* GapR protein only (Supplementary Figure S5A), without the detection of any diffraction from DNA. This dimer structure is also quite similar to the crystal structure 6CFY of free *Bosea* GapR (Supplementary Figure S5C). A comparison of this GapR dimer structure with that of the dimer-unit from the open tetramer complex revealed that the backbone heavy atom RMSD for residues 16–65 is 0.32 Å, while residues 66–89 show larger deviations for both protomers (Supplementary Figure S5B). SAXS analysis results indicated that the experimental Rg value of the free *Caulobacter* GapR in solution is 28.6 Å, much larger than the calculated Rg (22.8 Å) based on this GapR dimer structure. Comparison of the calculated scattering curves from the GapR dimer structure and the experimental SAXS data of the free GapR shows a fitting χ^2^ value of 21.9 (Figure 3E), indicating the solution structure of free GapR can not be like this dimer structure.

The fact that we got an open tetramer and a dimer structures for *Caulobacter* GapR, and the free *Bosea* GapR also produced a dimer structure (7), may suggest that the closed conformation of GapR is dynamic and its tetrameric interfaces could dissociate transiently.

### Free GapR is in equilibrium between open and closed tetrameric conformations

**Figure 5.**
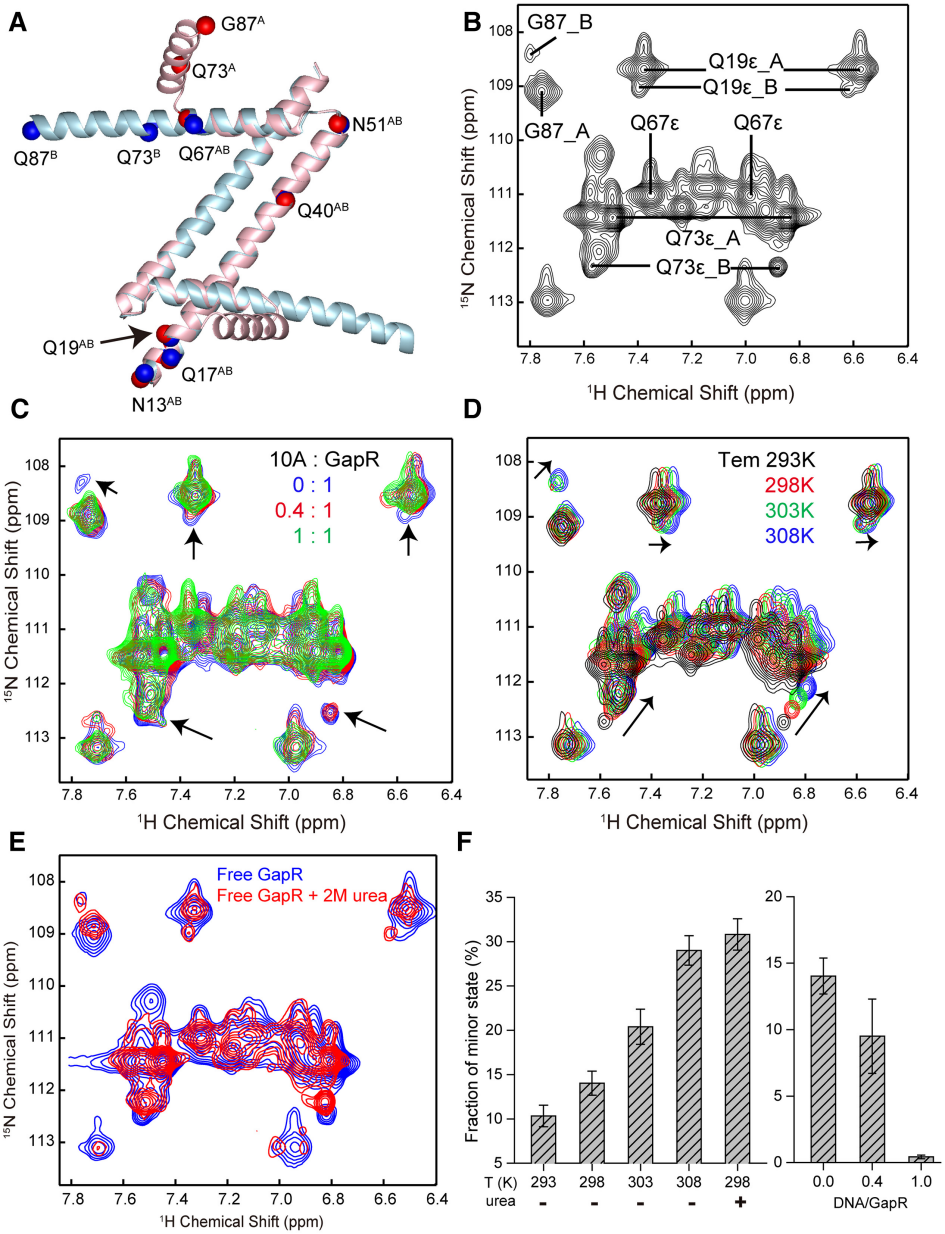
NMR analysis indicates free GapR adopting both major and minor tetrameric conformations in equilibrium. (**A**) Comparison of structures of dimer-units between open (cyan) and closed (pink) conformations of GapR. Corresponding backbone nitrogen atoms of glutamine and asparagine residues with side-chain NH_2_ groups and G87 are shown in red (letter A) or blue (letter B) balls, respectively, with the residue type and number indicated. (**B**) Selected region of 2D ^1^H–^15^N HSQC spectrum of free GapR showing signals from major (letter A) and minor (letter B) conformations for residues Q19, Q73, and G87. Assignments are indicated by the residue type and number. (**C**) Overlay of the selected region of 2D ^1^H–^15^N HSQC spectra of free GapR (blue) and with the addition of 10A DNA at DNA/protein ratios of 0.4 (red) and 1.0 (green), showing the disappearance of minor signals (indicated by arrows) upon DNA binding. (**D**) Overlay of the selected region of 2D ^1^H–^15^N HSQC spectra of free GapR at temperatures of 293 K (black), 298 K (red), 303 K (green), and 308 K (blue), showing the temperature-dependent intensity change of minor signals. (**E**) Overlay of the selected region of 2D ^1^H–^15^N HSQC spectra of GapR without (blue) and with (red) 2 M urea, showing the increase of minor signal intensities in the presence of urea. (**F**) The fractions of minor conformation of free GapR at different temperatures or in the presence of 2 M urea (left), and with the addition of 10A DNA (right). Fractions were calculated as an average based on major and minor signal intensities from residues Q19, Q73 and G87.

As the numbers of peak counts for 2D ^1^H–^15^N HSQC spectra of the free GapR or its complexes with DNAs are comparable to the number of residues of GapR, the four protomers of GapR tetramer should be symmetrical in solution. This is consistent with the closed tetramer structures of GapR (PDB ID: 6CG8 and 6OZX) (7,8), but not the open tetrameric structure. However, when we performed DNA binding study with 2D ^1^H–^15^N HSQC experiment, we noticed that there are several very weak peaks gradually disappeared as DNA is titrated in the GapR sample (Figure 5C and F).

As the most obvious minor peaks are consisted of two pairs of NH_2_ signals and one near a glycine NH signal, we tried to assign these signals by mutagenesis and generated four GapR mutants GapR^Q19S^, GapR^Q67S^, GapR^Q73S^ and GapR^G87A^. Comparison of 2D ^1^H–^15^N HSQC spectra of WT GapR and the mutants indicated that the two pairs of the minor NH_2_ signals are from residues Q19 and Q73, while the other minor peak is from residue G87 (Figure 5B; Supplementary Figure S6A-D). Temperature-dependent 2D ^1^H–^15^N HSQC spectra revealed that the intensities of these minor peaks are increased at higher temperatures, and decreased at lower temperatures (Figure 5D and F). The addition of 2M urea to GapR sample also results in an increase of the relative intensities of the minor peaks (Figure 5E and F). These indicate that the minor signals should be from a minor conformation of free GapR, which is in slow exchange with the major closed conformation. Upon DNA binding, the minor conformation is converted into the major conformation, since the minor peaks are disappeared for DNA bound GapR sample.

Considering that we have obtained crystal structures for an open tetramer and a dimer of GapR, we first examined the possibility that the minor conformer could be the dimer. Since we were unable to detect any sign of a dimer in free GapR in our molecular weight characterization, we wondered if there exists a dimer form which is in fast exchange equilibrium with the tetramer, and thus is inseparable from the tetramer. If this is the case, it is expected to observe a concentration-dependence for the experimental determined effective molecular weight. However, as shown in Figure 2B and D, the molecular weight values determined for free GapR samples of different concentrations are essentially the same, using both AUC and SEC-MALS techniques. In addition, the experimental Rg values we got for GapR samples at 1.0 and 2.0 mg/ml concentrations from SAXS analysis, are also virtually the same (28.6 and 28.8 Å), indicating that there is no concentration-dependent change for the size and shape.

We also investigated the possibility of obtaining dimeric protein by destabilizing the tetrameric interface of the closed tetramer through mutagenesis. Analysis of the structures of the closed tetramer reveals that residue Y82 is positioned in the hydrophobic core of the tetrameric interface, and residue E88 forms inter-protomer salt bridges with residues R65 and R69 (Supplementary Figure S7A), while residue D68 forms N-cap to stabilize the third α-helix in the closed tetramer (7). We thus generated three mutants GapR^D68A^, GapR^Y82A^ and GapR^ΔC3^ (removal of residues 87–89). Unfortunately, there was no soluble protein expressed for these three GapR mutants (data not shown). Analysis of the GapR dimer structure, it is apparent that the hydrophobic areas (residues 78–87) on the second α-helices are fully exposed (Supplementary Figure S7B), while the same hydrophobic areas in the closed tetramer participate in the formation of the tetrameric interface. It is possible that the dimer structure can not stably exist alone due to hydrophobic aggregation, when the C-terminal hydrophobic areas are fully exposed due to the disruption of the tetrameric interfaces for GapR^ΔC3^, GapR^D68A^ and GapR^Y82A^ mutants. For comparison, GapR^ΔC8^ and GapR^ΔC17^ are solubly expressed as a dimer, with the removal of C-terminal residues covering the hydrophobic areas.

As mentioned above, the closed tetrameric GapR/DNA complex structure agrees very well with the SAXS data of GapR in complexes with 11AT DNA, suggesting that the DNA bound GapR is in the closed conformation. This also agrees with the observation that the minor NMR signals are all converted to major signals when DNA is titrated in. Consistently, the experimental Rg values of the GapR/DNA complex in solution are quite similar to the calculated *R*_g_ value based on the crystal structure 6CG8. However, the experimental *R*_g_ value (28.6 Å) of the free GapR in solution is significantly larger than the calculated *R*_g_ value (26.9 Å) based on the crystal structure of the closed tetrameric protein, which should be the major conformer of free GapR. This difference in *R*_g_ values is, therefore, very likely due to the fact that the experimental *R*_g_ value for free GapR is actually the apparent radius gyration for a mixture of major and minor conformers, since SAXS measurements theoretically reflect the ensemble-averaged structures for molecules in solution. For a mixture sample of major and minor conformers, the apparent radius of gyration can be expressed as the square root of the weighted sum of the *R*_g_^2^ for each conformer:

$$\begin{equation*}\ R{{\rm g}_{{\rm app}}} = \sqrt {\left( {{C_{\rm A}}Rg_{\rm A}^2 + {C_{\rm B}}Rg_{\rm B}^2} \right)/\left( {{C_{\rm A}} + {C_{\rm B}}} \right)} \end{equation*}$$

where *C*_A_ and *C*_B_ are the mass concentration of the major and minor conformers with radii of gyration of *R*_gA_ and *R*_gB_, respectively (19–21). If the minor conformer is a dimer, then its radius gyration *R*_gB_ (22.8 Å) is even smaller than *R*_gA_ (26.9 Å) of the major conformer (closed tetramer), which can only result in a *R*_gapp_ that is smaller than *R*_gA_ based on the above equation. For *R*_gapp_ to be larger than *R*_gA_, *R*_gB_ has to be larger than both *R*_gA_ and *R*_gapp_. Taken together, the possibility can be ruled out for the closed tetramer to dissociate into dimer in free GapR.

We then investigated whether the minor conformer could be the open tetramer. The calculated *R*_g_ for the open tetrameric structure of GapR is 32.2 Å, much larger than that of the closed tetramer. Therefore, if the free GapR is a mixture of closed tetramer as the major conformer and open tetramer as the minor conformer, the apparent radius gyration *R*g_app_ would definitely be larger than *R*g_A_ of the major conformer. Also, as both the major and minor conformers are tetrameric, their exchange equilibrium constant should not be dependent on protein concentration, which is consistent with our observations that the experimentally measured molecular weight values do not vary with protein concentration (Figure 2B and D).

Analysis of the structural difference between open and closed tetramers reveals that two residues (Q19 and Q73) with side-chain NH_2_ minor signals should have significant different local chemical environments between the two conformations, along with residue G87 (Figure 5A; Supplementary Figure S8). The minor signals of residues Q73 and G87 in the 2D ^1^H–^15^N HSQC spectra of free GapR are significantly deviated from the corresponding major signals in position, while the minor signals of residue Q19 are partly overlapped with corresponding major signals, consistent with the fact that the structural difference between the open and closed tetramers for residue Q19 is smaller than those of residues Q73 and G87 (Figure 5A). It is interesting to notice that the relative intensities of minor peaks are increased in GapR^Q67S^ and GapR^G87A^ mutants, while the minor peaks are even stronger than the corresponding major peaks for GapR^G87A^ mutant (Supplementary Figure S6F). However, when DNA was titrated into GapR^G87A^ mutant, the minor peaks start to become weaker and eventually disappear, similar to that observed for the wild type protein (Supplementary Figure S6E and F). Also, it was noticed that the NMR samples of both GapR^Q67S^ and pR^G87A^ mutants precipitate much faster than the wildtype protein. As residues Q67 and G87 are located at critical positions of the tetrameric interface of the closed tetrameric GapR conformation, these observations are in line with that the minor conformation is involved in an opening of the tetrameric interface of the closed conformation.

**Figure 6.**
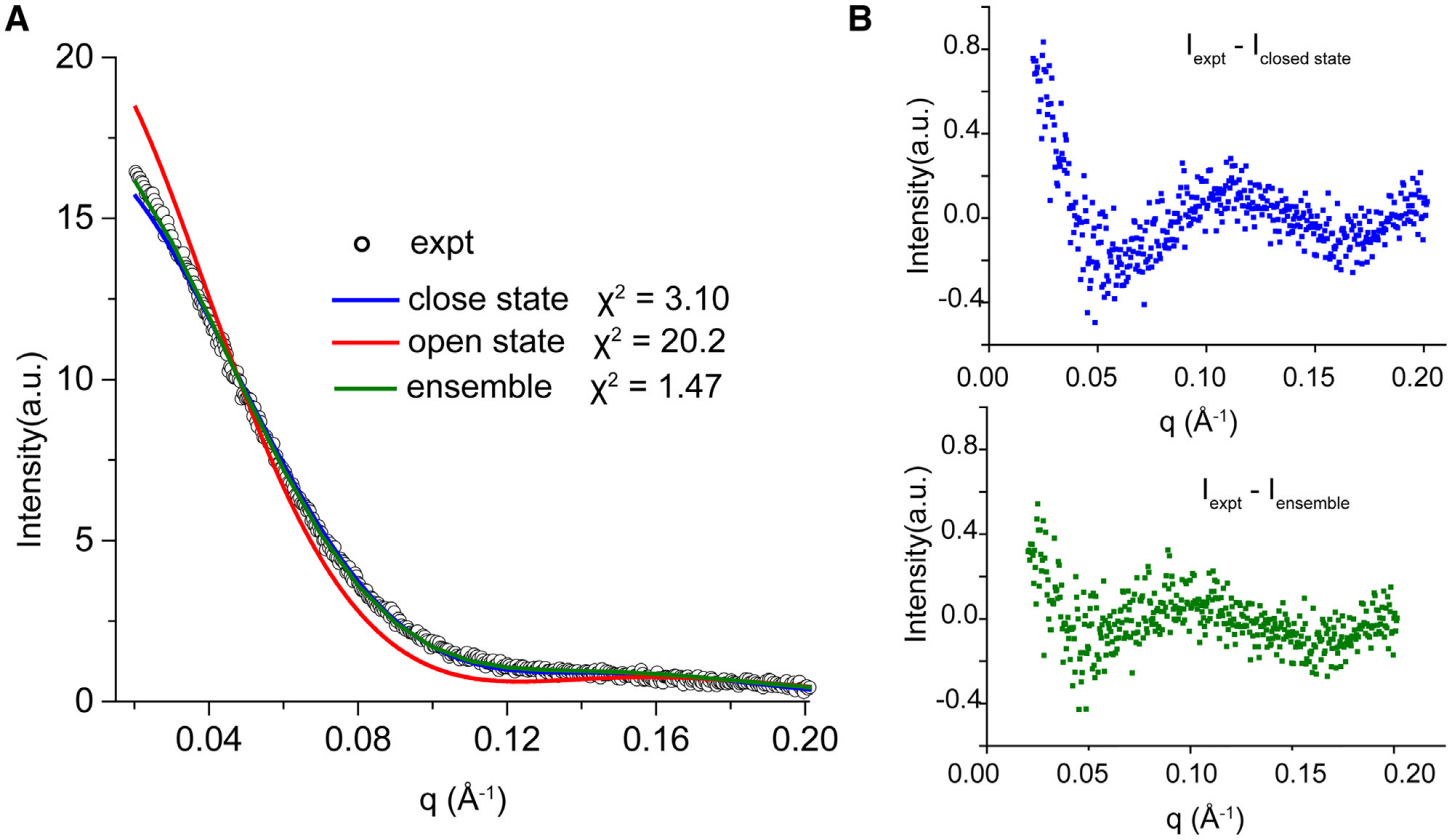
SAXS analysis suggests free GapR adopting both major closed conformation and minor open conformation. (**A**) Comparison of experimental SAXS data (gray dots) and calculated scattering curves from open (red line) and closed (blue line) conformations of GapR, and from a linear combination (open:closed = 12.5:87.5) of the two (green line). χ^2^ values between experimental data and each calculated curve are indicated. (**B**) Scattering intensity differences between experimental data of free GapR and calculated values from the closed conformation (upper) or the linear combination of open and closed conformations (lower) of GapR.

Furthermore, fitting of the experimental SAXS data of free GapR to a linearly combined scattering data calculated based on the open (12.5%) and closed (87.5%) tetramer structures produced a χ^2^ value of 1.47, much better fit than that of closed tetramer structure alone (χ^2^ of 3.10) (Figure 6) (22,23). Consistently, the averaged relative population of the minor state calculated based on peaks intensity ratios from NMR data is ∼14% at 298 K (Figure 5F), quite close to 12.5% of the minor state determined from SAXS data. These suggest that the minor conformation of GapR should be consistent with our newly determined open tetramer structure.

Taken all together, there exist both major conformation and minor conformation for free GapR in solution, which are in dynamic exchange equilibrium with each other. GapR binds DNA through the minor open conformation and is subsequently converted into the closed conformation, which fully locks the DNA inside its central tunnel (Figure 1B). Interestingly, Tarry *et al.* also proposed the possibility that GapR would open the closed conformation to bind DNA as a tetramer (8).

Although the SAXS and mutagenesis results indicate that the minor conformation of the free GapR is consistent with the structure of the open tetrameric GapR, we believe that the observed minor conformation should be from an ensemble of conformers with different opening scales for the two dimer-units. As the10A DNA is still partially surrounded by GapR in the open tetrameric structure, it is expected that further opening up of GapR should be required for DNA to enter the binding pocket. Therefore, the crystal structure of the open tetrameric GapR should be considered as representative of an ensemble of dynamic minor conformations, which should be in fast exchange on the NMR time scale.

### GapR binding widens the minor groove of DNA

We used isothermal titration calorimetry (ITC) to evaluate the binding affinities of GapR towards six different DNA sequences (Table 2; Supplementary Figure S9). The results showed that GapR indeed exhibits the highest affinity towards long stretch (≥10 bp) of continuous AT-rich sequences containing DNA (10A, 11AT and 5ApT), with similar *K*_d_ values of about 12 nM. For seq_1 and seq_2 sequences, which include 4 or 5 base pairs of A-tract sequences with lower AT contents, the affinities are lower with *K*_d_ of about 70 nM. Surprisingly, we found that GapR can also bind GC rich DNA sequence (6CpG) with a reasonable affinity of about 120 nM, although it is 10 times lower than that of AT-rich sequences. In addition, we observed almost the same NMR signal perturbation patterns for GapR titration with 6CpG and 10A DNA sequences, in both 2D ^1^H–^15^N HSQC and 2D ^1^H–^13^C HSQC spectra (Supplementary Figure S10), indicating that GapR binds both AT-rich and GC-rich DNA through the same binding mode.

**Table 2.** Equilibrium dissociation constants and thermodynamic parameters for GapR to bind different duplex DNA sequences.

	Sequence	*K*_d_(nM)	Δ*H* (kJ/mol)	Δ*TS* (kJ/mol)	Δ*G* (kJ/mol)
10A	F: 5′-CCGAAAAAAAAAACGC	11.6 ± 2.5	46.2 ± 0.4	88.4	–42.2
	R: 5′-GCGTTTTTTTTTTCGG				
11AT	F: 5′-TTAAAATTAAA	12.3 ± 4.5	32.2 ± 0.5	74.3	–42.0
	R: 5′-TTTAATTTTAA				
5ApT	F: 5′-CCGATATATATATCGC	12.3 ± 8.9	11.1 ± 0.2	53.2	–42.0
	R: 5′-GCGATATATATATCGG				
5ApT+NT	F: 5′-CCGATATATATATCGC	16.6 ± 4.5	24.5 ± 0.2	65.8	–41.4
	R: 5′-GCGATATATATATCGG				
seq1	F: 5′-GCGAAATTGATCG	72 ± 24	30.6 ± 0.5	68.6	–38.0
	R: 5′-CGATCAATTTCGC				
seq2	F: 5′-CGTGTTTTCGG	72 ± 19	30.2 ± 0.4	68.2	–38.0
	R: 5′-CCGAAAACACG				
6CpG	F: 5′-CGCGCGCGCGCG	118 ± 17	35.9 ± 0.4	72.8	–36.8
	R: 5′-CGCGCGCGCGCG				

The combined standard deviations were calculated from two measurements. (NT: netropsin)

The thermodynamic parameters determined from ITC analysis indicate that the DNA binding process of GapR is endothermic and entropy-driven (Δ*H*° > 0, –*TS*° < 0), suggesting that GapR binds DNA non-specifically and mainly through electrostatic interactions (24,25). Among the three long stretch AT-rich DNA sequences, 5ApT DNA (with four consecutive TpA steps) has the lowest enthalpic cost (11.1 kJ/mol) for GapR to bind, while 10A DNA (with 10-bp A-tract) has the highest enthalpic cost (46.2 kJ/mol), over four times of that of 5ApT. In addition, 11AT DNA with a 6-bp A-tract flanked by two TpA steps, has a binding enthalpy of 32.2 kJ/mol. It is well established that A-tract DNA sequences are structurally rigid and normally have a narrower minor groove among AT-rich DNAs (26,27). However, DNA sequences with multiple TpA steps are most flexible and have larger variations in global and local conformations (14), which can be turned into A-tract like conformation and become more rigid, due to the binding of minor groove binding agent netropsin (28,29). Thus, we analyzed the binding of GapR towards the netropsin bound 5ApT sequence, and the results showed that netropsin binding indeed increases the enthalpic cost (24.5 kJ/mol) by over 2-fold for 5ApT DNA to bind GapR, while still retains similar binding affinity. These may hint that the large difference in binding enthalpy between 10A and 5ApT sequences is likelyresulted from the deformation of DNA upon binding GapR.

**Figure 7.**
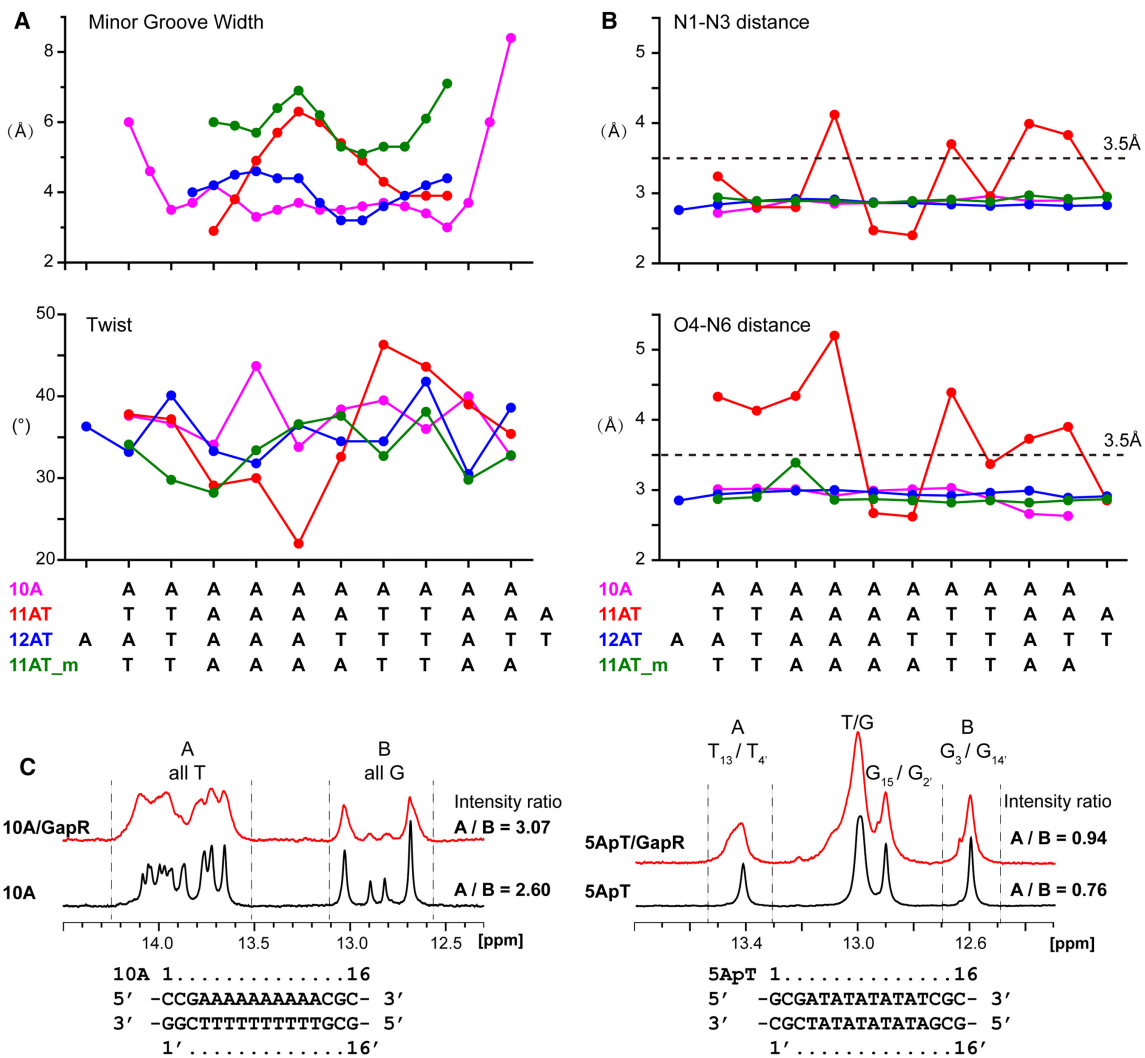
GapR binding widens the minor groove of DNA. (**A**) Comparison of the minor groove width and twist values of GapR bound 10A DNA and 11AT DNA from crystal structure 6CG8 with (11AT_m) and without energy minimization, along with free 12AT DNA (PDB ID: 4J2I). (**B**) Comparison of acceptor–to-donor N1-N3 and O4-N6 distances of the base pair hydrogen bonds for GapR bound 10A DNA and 11AT DNA from crystal structure 6CG8 with (11AT_m) and without energy minimization, along with the free 12AT DNA (PDB ID: 4J2I). (**C**) Comparison of 1D imino ^1^H NMR spectra of 10A (left) and 5ApT (right) DNAs with (red) and without (black) GapR binding. Tentative assignments of DNA imino proton signals are indicated. The imino proton signal intensity ratios between thymines and guanines are indicated.

Comparing the DNA conformations of 11AT and 10A DNAs in the protein/DNA complex structures, it is found that both the minor and major groove widths of the closed tetrameric GapR bound 11AT DNA are wider than those of the open tetrameric GapR bound 10A DNA, for the middle part of the DNAs (Figure 7A; Supplementary Figure S11). As there is no structure for free 11AT DNA, we used the structure of a free 12-bp AT-rich DNA sequence (PDB ID: 4J2I) which also has a 6-bp A-tract sequence flanked by two TpA steps (30), for comparison. Indeed, both minor groove and major groove widths of 12AT DNA are closer to those of 10A than 11AT DNA (Figure 7A; Supplementary Figure S11). Besides, 11AT DNA displays undertwist for the first half, but overtwist for the second half (Figure 7A; Supplementary Figure S11), while the twist values of 10A and 12AT DNAs are around standard value (36°) for B-form DNA (31). Analysis of base pair geometry parameters revealed that the opening values of 11AT DNA are significantly larger than those of 10A and 12AT DNAs for most base pairs, and the opening values are even over 20°for the first 4 base pairs (Supplementary Figure S11). As large base pair opening values may indicate the break of base pair hydrogen bonds, we analyzed the base pair hydrogen bonding patterns for 11AT, 12AT and 10A DNAs (Figure 7B), using an acceptor-donor distance cutoff of 3.5 Å (32). The results showed that 12AT and 10A DNAs have standard Watson-Crick base pairs. However, for 11AT DNA, four base pairs no longer exist, while three of the base pairs become non-Watson–Crick with only one hydrogen bond of N1–N3. Therefore, the double helix of 11AT DNA is highly distorted in the crystal structure 6CG8 of the closed conformation, as a number of base pair hydrogen bonds are broken.

We then used 1D ^1^H-NMR spectroscopy to assess the stability of base pairs of GapR bound DNA by monitoring the imino proton signals of thymine and guanine, and tentatively assigned the imino proton signals of the free 10A and 5ApT DNAs based on chemical shift prediction from DSHIFT (33). Compared with those of the free DNA, the imino protons signals are broadened as expected for GapR bound 10A or 5ApT DNA, while the signal chemical shifts and the peak distribution patterns are still quite similar (Figure 7C). The chemical shifts of guanine imino protons are barely changed, consistent with that GapR mainly binds the 10-bp AT rich region of DNA, while the GC base pairs should be mostly free of protein contact. Importantly, the imino proton signal intensity ratios between thymines and guanines become larger when DNA is bound by GapR (Figure 7C), consistent with that the imino protons of thymine should be protected from the solvent exchange due to GapR binding. These observations do not support that AT base pair hydrogen bonds are broken in GapR bound DNA, which would result in a decreased signal intensity ratio, since the imino protons of thymines and guanines are invisible by 1D ^1^H NMR without forming base pair hydrogen bonds.

Next, we carried out an energy minimization for the GapR/11AT complex structure of 6CG8 using AMBER12 (34), and re-analyzed the conformation and geometry of 11AT DNA in this energy minimized complex structure. The results show that all base pair hydrogen bonds become normal for 11AT DNA after energy minimization (Figure 7B), while the opening and twist parameters are also comparable to those of 12AT and 10A DNAs (Figure 7A; Supplementary Figure S11). Remarkably, we found that the minor groove of 11AT DNA is widened significantly in the energy minimized 6CG8 structure, with the minor groove widths uniformly larger than those of 12AT and 10A DNAs by 1–3 Å (Figure 7A). This can explain why GapR binds A-tract 10A DNA with much higher enthalpy cost than that of 5ApT DNA, as A-tract DNAs are rather rigid and their minor groove widths usually are below 5 Å. It has been demonstrated that protein binds DNA with a net unfavorable ΔH as the result of molecular strain, when DNA is strongly distorted (35). Therefore, it is expected that the enthalpy cost would be much higher for GapR binding to widen the rigid narrow minor groove of A-tract DNA, than that of the flexible consecutive TpA steps containing DNA.

In addition, the protein–DNA contact interface in the energy minimized structure is essentially the same as that in the crystal structure 6CG8, except that three additional K71 side-chains are positioned within 5.5 Å from phosphate groups of the DNA (Supplementary Figure S4C). Almost all the distances between DNA phosphate groups and corresponding side-chains of positively charged residues become shorter after energy minimization.

## DISCUSSION

GapR is an essential nucleoid-associated protein highly conserved throughout the α-proteobacteria (2). Our current studies clearly demonstrate that free GapR is a tetramer, but not a dimer. We have used multiple techniques to analyze the oligomerization and the molecular weight of free GapR, and there is no indication at all for the existence of dimeric GapR. We have shown that free GapR adopts multiple conformations in dynamic equilibrium, while the major conformer of free GapR should adopt a closed conformation resembling those in the structure of the GapR/11AT complex (7) or the newly reported crystal structures of GapR (8). We propose that the minor conformation we observed should be from an ensemble of conformers with different openings of the two dimer-units, and the open tetrameric GapR structure is a representative of multiple dynamic minor conformations in fast exchange.

Although it was initially reported that free GapR exists as a dimer which can form a tetramer (dimer-of-dimers) upon binding DNA and encircle the overtwisted DNA (7), our study results and those from Tarry *et al.* all indicate that the free GapR is a tetramer (8). A most recent study published while this paper was under review also showed that the free GapR exists as a tetramer (36). We have not observed any indication for the minor conformers of the free GapR to be a dimer either. We suspected that GapR in the dimeric conformation inclines to aggregate due to the expose of C-terminal hydrophobic regions, as indicated by that several mutations disrupting the closed tetrameric interfaces results in insoluble protein expression in *E. coli*, while soluble dimeric proteins could be obtained with the removal of C-terminal hydrophobic residues. Besides, this is also consistent with that the free GapR tends to precipitate, but not DNA bound GapR. The partially opening of the closed tetrameric interfaces of the free GapR also involves transiently exposing the C-terminal hydrophobic areas. We also observed faster precipitation for the GapR^Q67S^ and GapR^G87A^ mutants, which have a higher relative population for the minor conformers.

Taken all together, we propose a new mechanism for GapR to bind DNA. The major closed conformation of GapR can open its tetrameric interface to become the minor conformers, which can easily recognize DNA and form a complex with DNA firstly in the open tetrameric conformation (Figure 1B). The bound DNA induces the open tetramer of GapR to undergo structural rearrangement, in which each of the four long α2 helices is kinked and becomes two short α helices linked by an ‘elbow’ consisted of residues Q67 and D68. The two short α helices form new tetrameric interfaces with another protomer, converting GapR into the closed tetrameric conformation. Our mutagenesis study results suggest that the ‘elbow’ and the C-terminal residues should play important roles in the dynamic opening of GapR structure. The interactions between GapR and DNA should stabilize the closed tetrameric conformation.

ITC analysis results reveal that GapR indeed displays binding selectivity towards long stretch of consecutive AT base pair containing DNA sequences (*K*_d_ ∼ 12 nM). However, GapR still binds all GC base pair containing DNA with a reasonable high affinity (*K*_d_ ∼ 120 nM), which can explain why GapR is also found to be enriched in regions with low AT contents, such as 3′ ends of highly expressed transcription units of tRNAs (7). Contrary to the previous report (7), our results indicate that GapR bound DNA does not show characteristics of overtwisting, nor narrower minor groove. Instead, we found GapR binding results in widening the minor groove of AT-rich DNA, which is supported by the binding enthalpy cost differences of GapR toward A-tract DNA and consecutive TpA containing DNA with or without bound netropsin. These are all consistent with the recent report that the central tunnel of the closed tetramer can be wide enough to accommodate B-DNA comfortably (8).

Furthermore, Tarry *et al.* proposed that GapR with a wider central tunnel would scan along B-DNA until it encounters AT-rich DNA, which would localize GapR at the higher affinity binding position. The fact that we only observed one methyl signal for M38 in 2D ^1^H–^13^C HSQC spectrum for GapR/DNA complex (Figure 3B; Supplementary Figure S10B), may suggest that the bound DNA inside the central tunnel of the closed tetramer does not adopt a unique relative position with the protein. Otherwise, we would expect to observe four different peaks for the methyl groups of M38 from the four protomers, since their distances to the nearest DNA atoms are quite different in the crystal structure 6CG8 (Supplementary Figure S12). In addition, Lourenco *et al.* reported that GapR can stimulate DNA bridging in vitro, which still can not be explicitly explained from the currently available structural study results (36). Therefore, more structural and functional investigations are required to further reveal the molecular mechanisms for the functions of GapR in DNA replication and transcription.

## DATA AVAILABILITY

PDBePISA server for the exploration of macromolecular interfaces is available at http://www.ebi.ac.uk/msd-srv/prot_int/cgi-bin/piserver.

The Curves+ software for analyzing the structure of nucleic acids is available at http://curvesplus.bsc.es/analyse.

Structures of GapR/10A complex and free dimeric GapR from *C. crescentus* have been deposited at the Protein Data Bank under accession numbers 6K2J and 6JYK, respectively.

## Supplementary Material

gkaa644_Supplemental_File
