# Supplementary material for: GapR binds DNA through dynamic opening of its tetrameric interface
Source: Nucleic Acids Res. 2020 Aug 5;48(16):9372–86. doi: 10.1093/nar/gkaa644 (PMC7498317; doi:10.1093/nar/gkaa644)
Supplement: gkaa644_Supplemental_File [file gkaa644_supplemental_file.pdf]

Supplementary Table and Figures

Table S1. Apparent molecular weight of GapR and its truncation mutants determined from SEC analysis.

Protein	Retention Volume (mL)	Calculated molecular weight* (kDa)	Theoretical molecular weight of monomer (kDa)
**GapR**	1.985	56.6	11.2
**$GapR^{\Delta N10}$**	2.020	50.0	9.4
**$GapR^{\Delta C17}$**	2.198	26.5	9.3
**$GapR^{\Delta C8}$**	2.137	32.9	10.3

*The molecular weight was calculated based on a calibration equation ($\log_{10}$[MW] = -1.5506 x [RV] + 4.8331; R^2 = 0.997) generated based on the retention volume (RV) values of standard proteins Ribonuclease A from bovine pancreas (13.7 kDa), Carbonic Anhydrase from bovine erythrocytes (29.0 kDa) Ovalbumin from hen egg (44.0 kDa), Conalbumin from chicken egg white (75.0 kDa), Aldolase from Rabbit muscle (158.0 kDa), and Thyroglobulin from bovine thyroid (669.0 kDa).

Figure S1. Chemical cross-linking analysis of GapR and GapR$^{\Delta C17}$ with different concentrations of EGS.

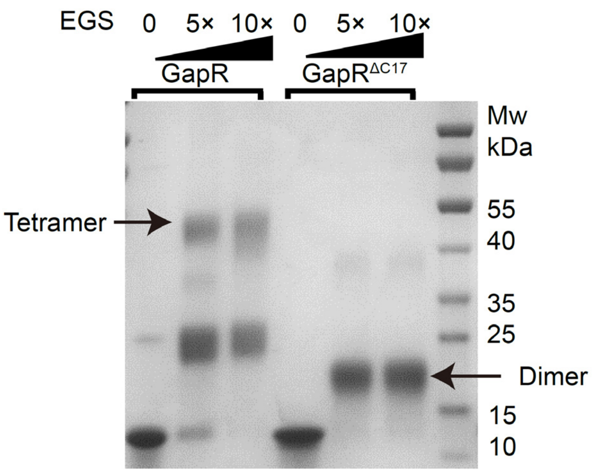

Figure S2. NMR analysis of GapR and its truncation mutants. (A) Overlay of 2D ^{1}H-^{15}N HSQC spectra of GapR (blue) and GapR $^{\Delta N10}$ (red). (B) Overlay of 2D ^{1}H-^{15}N HSQC spectra of GapR (blue) and GapR$^{\Delta C17}$ (red). (C) Overlay of 2D ^{1}H-^{15}N HSQC spectra of GapR$^{\Delta C17}$ (red) and GapR$^{\Delta C8}$ (blue).

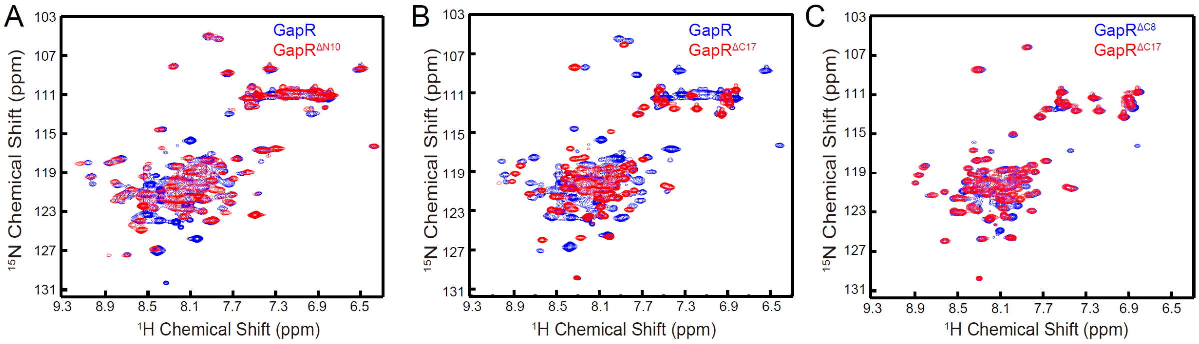

Figure S3. Dimer interface of the dimeric unit (A) and tetramer interface (B) of GapR in the crystal structure of the open GapR/10A complex (PDB ID: 6K2J), with sidechains of residues forming the hydrophobic pocket and residues forming salt bridges shown as ball and stick. (C) Comparison of the two protomers in a dimeric unit.

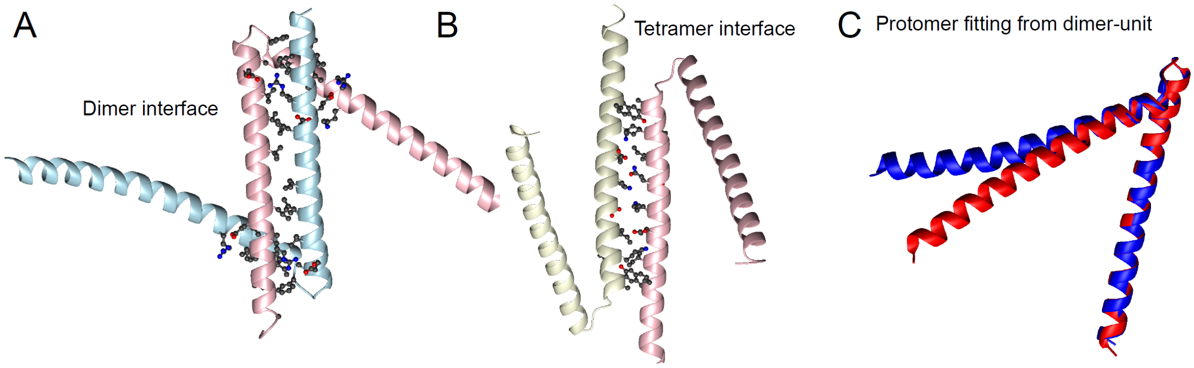

Figure S4. Analysis of electrostatic interactions between GapR and DNA for crystal structures of GapR/10A (PDB ID: 6K2J) (A) and GapR/11AT (PDB ID: 6CG8) without (B) and with (C) energy minimization. The shortest distances between positively charged lysine and arginine sidechain nitrogen atoms and corresponding oxygen atoms of DNA phosphate groups are indicated, with a distance cutoff of 6.5 Å. Hydrogen bonds of DNA base pairs are indicated by arrows, and red X is used to indicate no hydrogen bond between bases. Residues are labeled by the amino acid type and number, with upper case "A, B, C, or D" for the indication of different protomers.

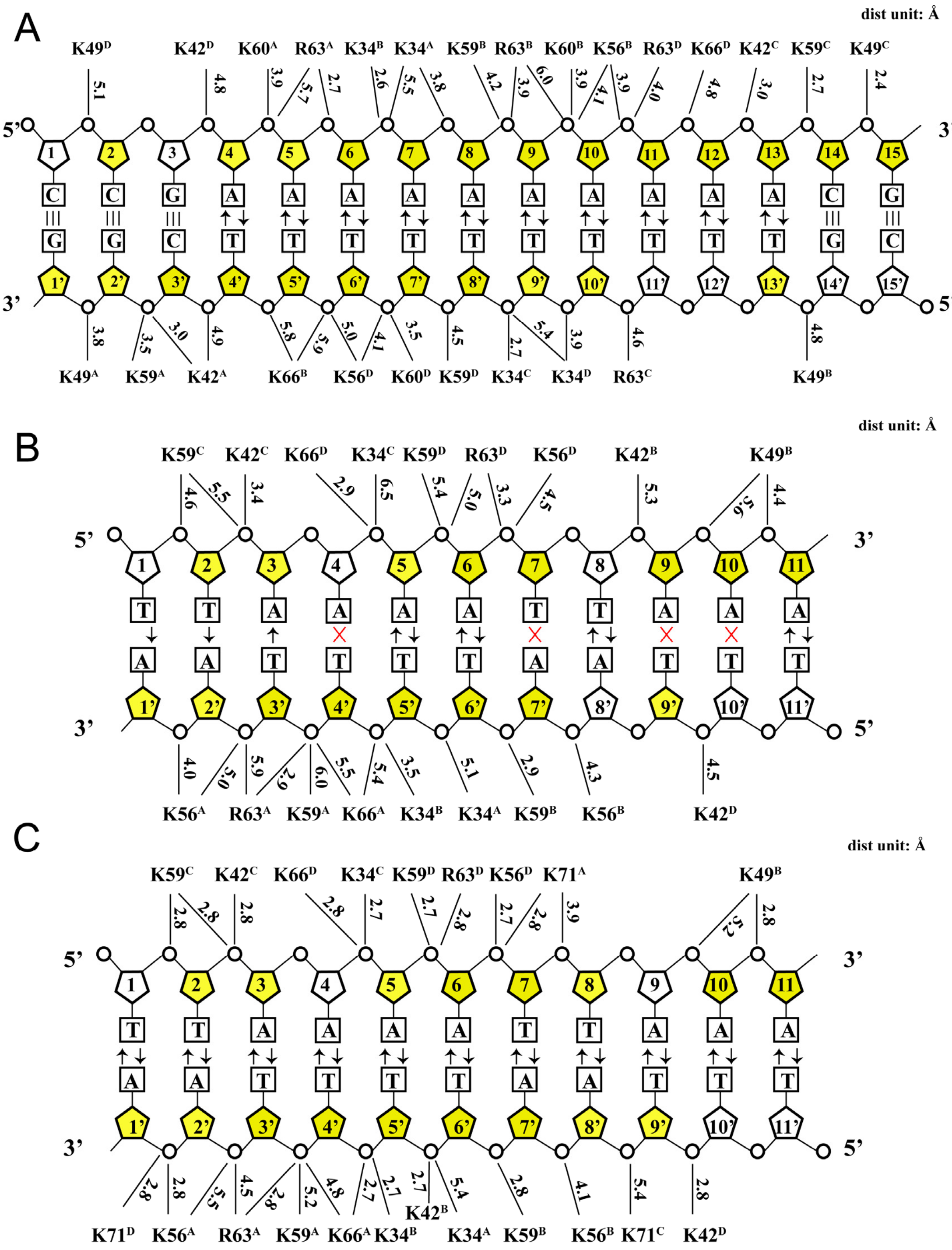

Figure S5. (A) Ribbon diagram of the crystal structure of GapR dimer (PDB ID: 6JYK). (B) Comparison of crystal structures of GapR dimer (blue) and GapR dimeric unit from crystal structure 6K2J (red). (C) Comparison of crystal structures of GapR dimer from *C. crescentus* (blue) and *Bosea sp. Root381* (gray).

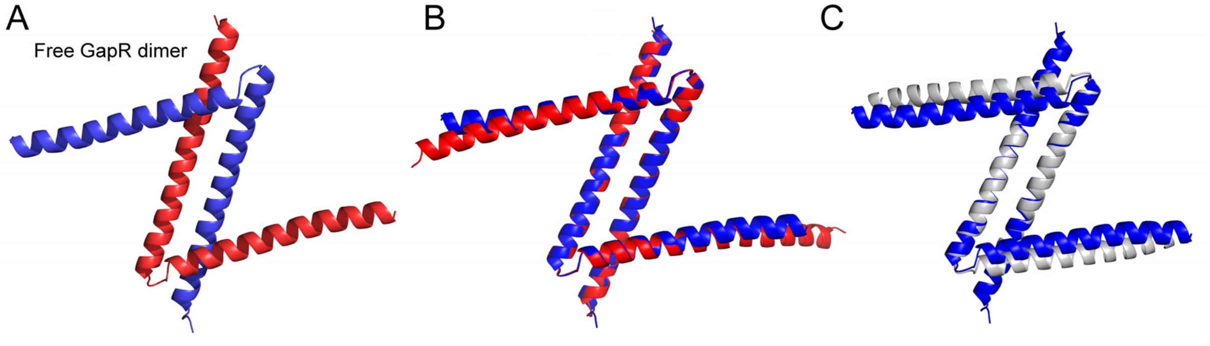

Figure S6. Overlay of selected regions of 2D ^{1}H-^{15}N HSQC spectra between WT GapR (blue) and its mutants (red) of (A) GapR^{Q19S}, (B) GapR^{Q67S}, (C) GapR^{Q73S}, (D) GapR^{G87A}. (E) Overlay of selected regions of 2D ^{1}H-^{15}N HSQC spectra of free (blue) and 10A DNA-bound GapR^{G87A} mutant (red for DNA:protein = 0.5:1; green for DNA:protein = 1:1). (F) The fractions of minor state for WT GapR and its mutants GapR^{Q67S} and GapR^{G87A}, along with 10A DNA-bound GapR^{G87A}.

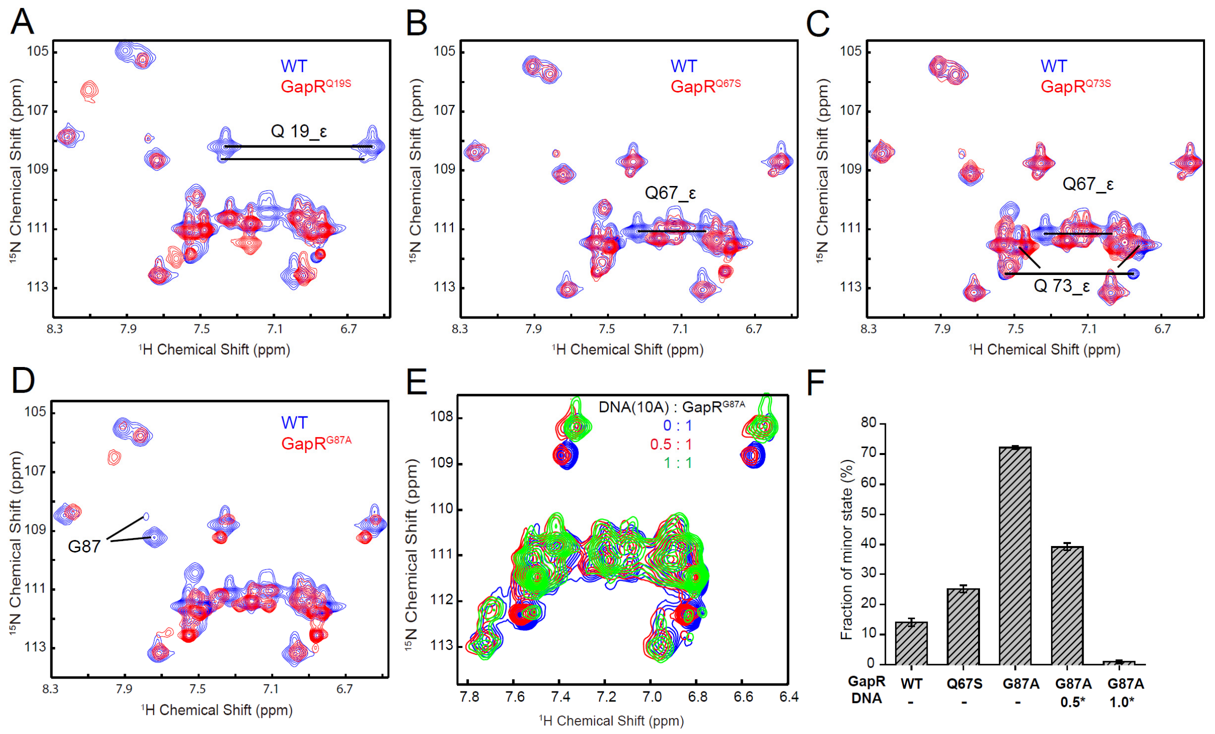

Figure S7. (A) Tetrameric interface of GapR in the closed GapR/11AT complex structure (PDB ID: 6CG8), with sidechains of residues forming the hydrophobic pocket and residues forming salt bridges shown as ball and stick. (B) Space filled diagram of the GapR dimer structure, with atoms from positively charged residues (blue), negatively charged residues (red), hydrophobic residues (gold), and polar residues (gray) shown as balls. Atoms of residue Y82 are indicated in green, and the hydrophobic areas on α2 helixes are indicated by yellow circles.

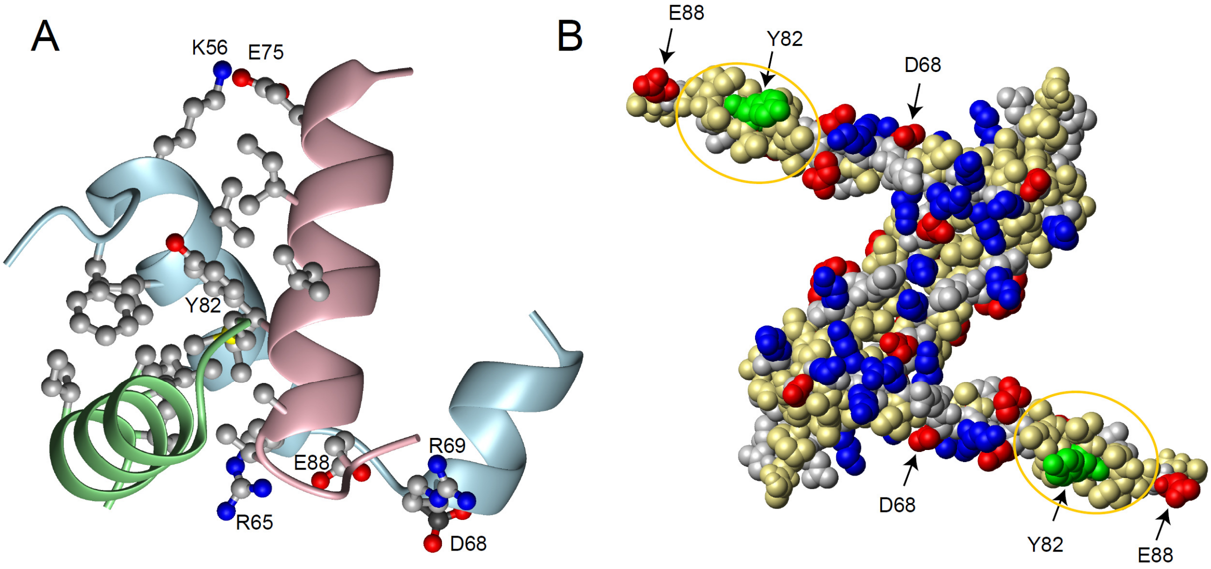

Figure S8. Structural comparison of the open and closed conformations of GapR. Ribbon diagrams of closed (A) and open (B) tetrameric conformations of GapR, with backbone nitrogen atoms of residues Q19 (yellow), Q67 (purple), Q73(green), and G87(red) shown as balls.

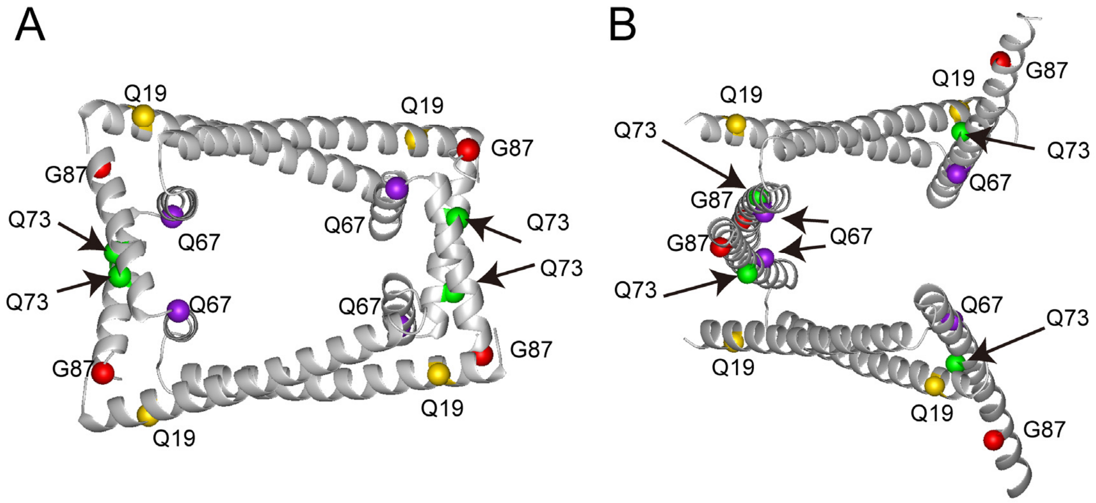

Figure S9. ITC results for GapR to bind DNA sequences 10A (A), 11AT (B), 5ApT (C), 5ApT with netropsin (D), Seq_1 (E), Seq_2 (F), and 6CpG (G).

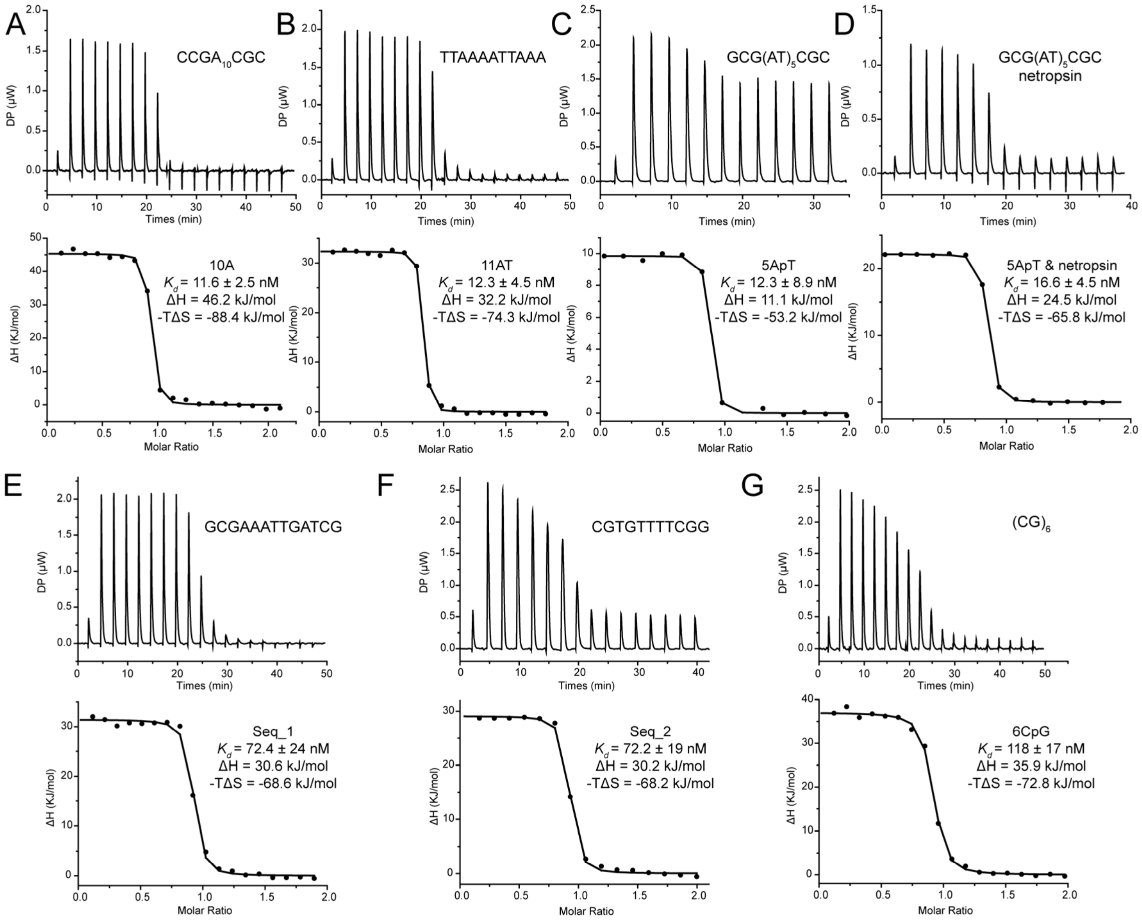

Figure S10. (A) Overlay of 2D ^{1}H-^{15}N HSQC spectra of free GapR (blue) and with the addition of 6CpG DNA at DNA/protein ratios of 0.4 (gold), 0.6 (red), and 1.0 (green). (B) Overlay of 2D ^{1}H-^{13}C HSQC spectra of free (blue) GapR and with the addition of 6CpG DNA at DNA/protein ratios of 0.4 (gold), 0.6 (red), and 1.0 (green). Signal of M38 sidechain methyl group is indicated by box.

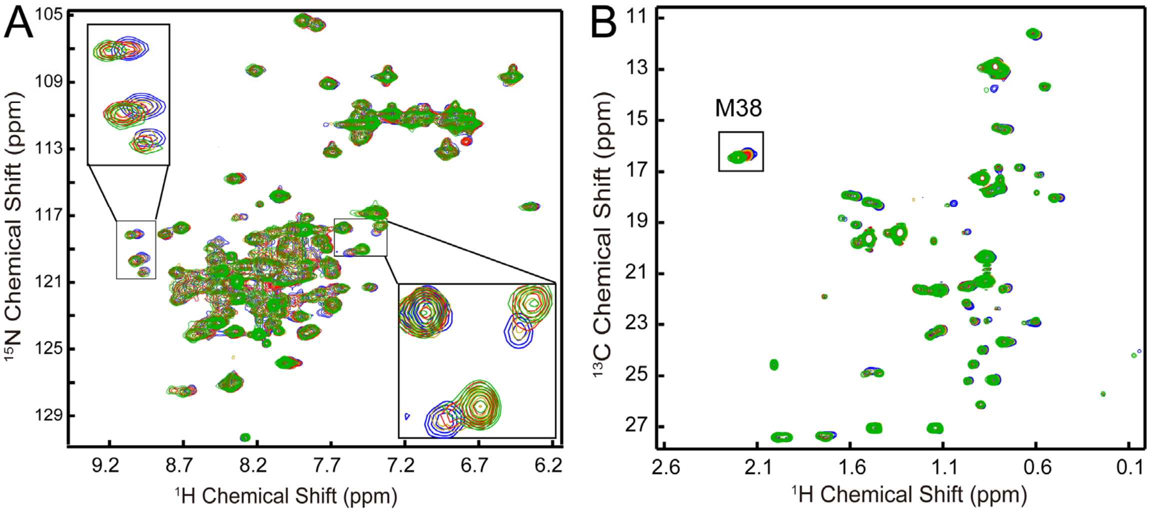

Figure S11. Comparison of the major groove width and opening values of GapR bound 10A DNA and 11AT DNA from crystal structure 6CG8 with (11AT_m) and without energy minimization, along with free 12AT DNA (PDB ID: 4J2I).

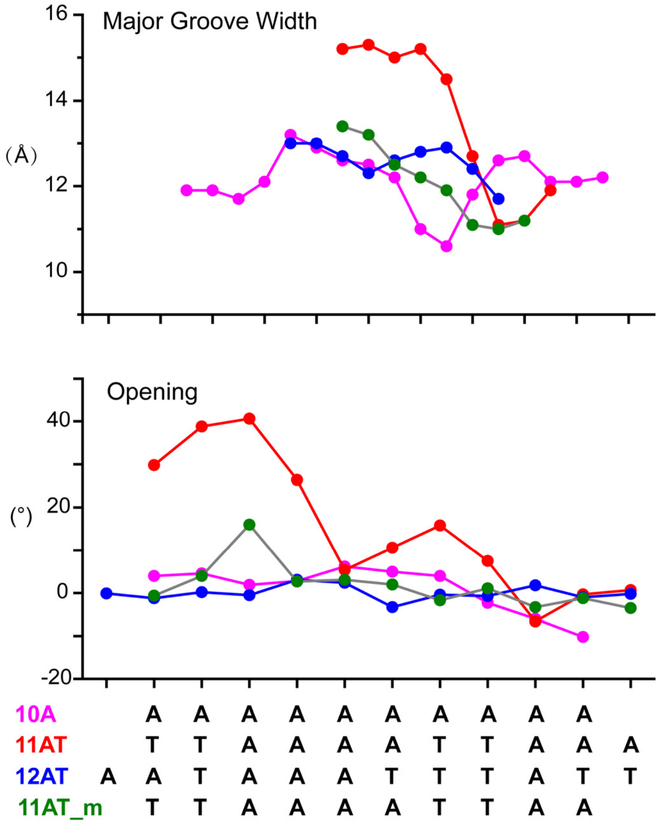

Figure S12. Ribbon diagrams of the closed GapR/11AT complex structure (PDB ID: 6CG8), with sidechains of residues M38 shown as ball and stick. The tetrameric GapR is colored in gray, while DNA is indicated in gold. The distances between methyl groups of residue M38 from the four protomers ($M38^A$, $M38^{B,}$ $M38^C$ and $M38^D$) and corresponding nearest (phosphorus) atoms of DNA are indicated in the table below, respectively.

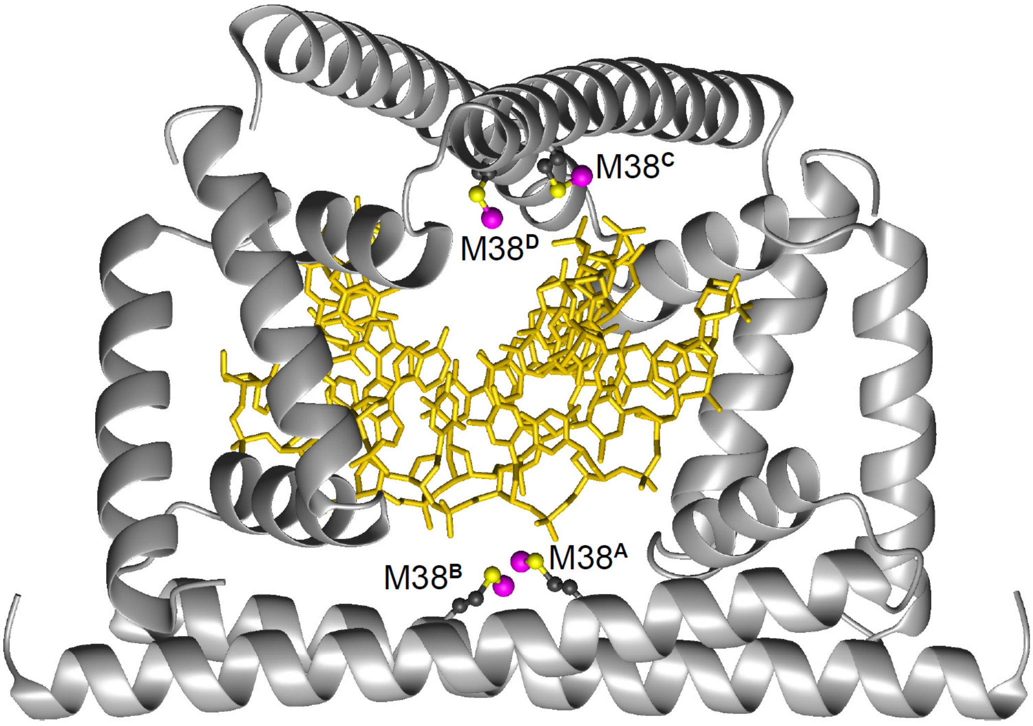

Distance	$M38^A$	$M38^B$	$M38^C$	$M38^D$
Nearest Atom of DNA	4.6 Å (R:8T:OP2)	4.7 Å (R:8T:OP1)	3.8 Å (F:4A:OP2)	6.9 Å (F:4A:C2')
Nearest P Atom of DNA	6.0 Å (R:8T:P)	5.4 Å (R:8T:P)	4.8 Å (F:4A:P)	9.3 Å (F:5A:P)
